# Dual-single-guide RNA strategy improves CRISPR-mediated homology-directed repair in *Aspergillus*

**DOI:** 10.1093/nar/gkag095

**Published:** 2026-02-05

**Authors:** Mingxin Fu, Jing Wang, Jingyi Li, Yao Zhou, Xiaofei Huang, Zehan Jia, Yiqing Luo, Xinyu Tan, Yan Gao, Bingzi Yu, Yuting Duan, Qianyun Bu, Xiaoying Li, Yifan Wang, Naoki Takaya, Shengmin Zhou

**Affiliations:** State Key Laboratory of Bioreactor Engineering, School of Biotechnology, East China University of Science and Technology, Shanghai 200237, China; State Key Laboratory of Bioreactor Engineering, School of Biotechnology, East China University of Science and Technology, Shanghai 200237, China; State Key Laboratory of Bioreactor Engineering, School of Biotechnology, East China University of Science and Technology, Shanghai 200237, China; State Key Laboratory of Bioreactor Engineering, School of Biotechnology, East China University of Science and Technology, Shanghai 200237, China; State Key Laboratory of Bioreactor Engineering, School of Biotechnology, East China University of Science and Technology, Shanghai 200237, China; State Key Laboratory of Bioreactor Engineering, School of Biotechnology, East China University of Science and Technology, Shanghai 200237, China; State Key Laboratory of Bioreactor Engineering, School of Biotechnology, East China University of Science and Technology, Shanghai 200237, China; State Key Laboratory of Bioreactor Engineering, School of Biotechnology, East China University of Science and Technology, Shanghai 200237, China; State Key Laboratory of Bioreactor Engineering, School of Biotechnology, East China University of Science and Technology, Shanghai 200237, China; State Key Laboratory of Bioreactor Engineering, School of Biotechnology, East China University of Science and Technology, Shanghai 200237, China; State Key Laboratory of Bioreactor Engineering, School of Biotechnology, East China University of Science and Technology, Shanghai 200237, China; State Key Laboratory of Bioreactor Engineering, School of Biotechnology, East China University of Science and Technology, Shanghai 200237, China; State Key Laboratory of Bioreactor Engineering, School of Biotechnology, East China University of Science and Technology, Shanghai 200237, China; State Key Laboratory of Bioreactor Engineering, School of Biotechnology, East China University of Science and Technology, Shanghai 200237, China; Faculty of Life and Environmental Sciences, Microbiology Research Center for Sustainability, Tsukuba Institute for Advanced Research, University of Tsukuba, Tsukuba, Ibaraki 305-8572, Japan; State Key Laboratory of Bioreactor Engineering, School of Biotechnology, East China University of Science and Technology, Shanghai 200237, China; State Key Laboratory of Natural and Biomimetic Drugs, Peking University, Beijing 100191, P R China

## Abstract

CRISPR–Cas9 knock-in efficiency is often limited by geometric misalignment between donor DNA and the endogenous strand-invasion path. In *Aspergillus nidulans*, we found that integration drops sharply when the insertion site is offset from the invasion entry point, producing premature annealing or unsupported 3′ ends that stall DNA synthesis. Chromatin immunoprecipitation-based profiling shows directional loading of the RAD51 homolog UvsC around Cas9-induced double-strand breaks, thereby defining the spatial origin of strand invasion. Guided by this insight, we introduce a dual-single-guide RNA design that places two cuts flanking the insertion site to create a geometry-matched strand-invasion window. This alignment consistently and markedly increases homology-directed-repair-mediated integration across insert sizes and editing tasks—including C-terminal tagging, bidirectional promoter rewiring, and long-distance dual-site mutagenesis—and generalizes across multiple fungal species. We propose a structural-docking model in which pairing fidelity between the resected chromosomal strand and donor homology arms governs knock-in outcomes, providing a practical design principle for efficient and precise genome engineering at structurally constrained loci.

## Graphical Abstract

**Graphical Abstract:**
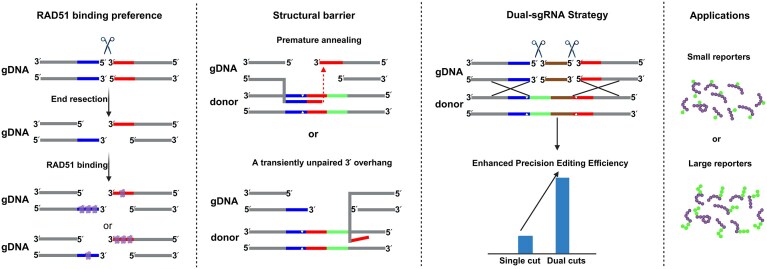

## Introduction

The CRISPR–Cas9 system has become a mainstream technology for precise genome editing in eukaryotic organisms [1]. A single-guide RNA (sgRNA) directs Cas9 to a specified locus to introduce a double-strand break (DSB), activating endogenous DNA-repair pathways [2]. A common strategy relies on homology-directed repair (HDR) using donor DNA as the template [3, 4]. Through recombinationmediated pairing and DNA synthesis, HDR supports targeted insertions [5], epitope tagging [6], promoter replacement [7], and multisite mutagenesis [8].

**Figure 1.**
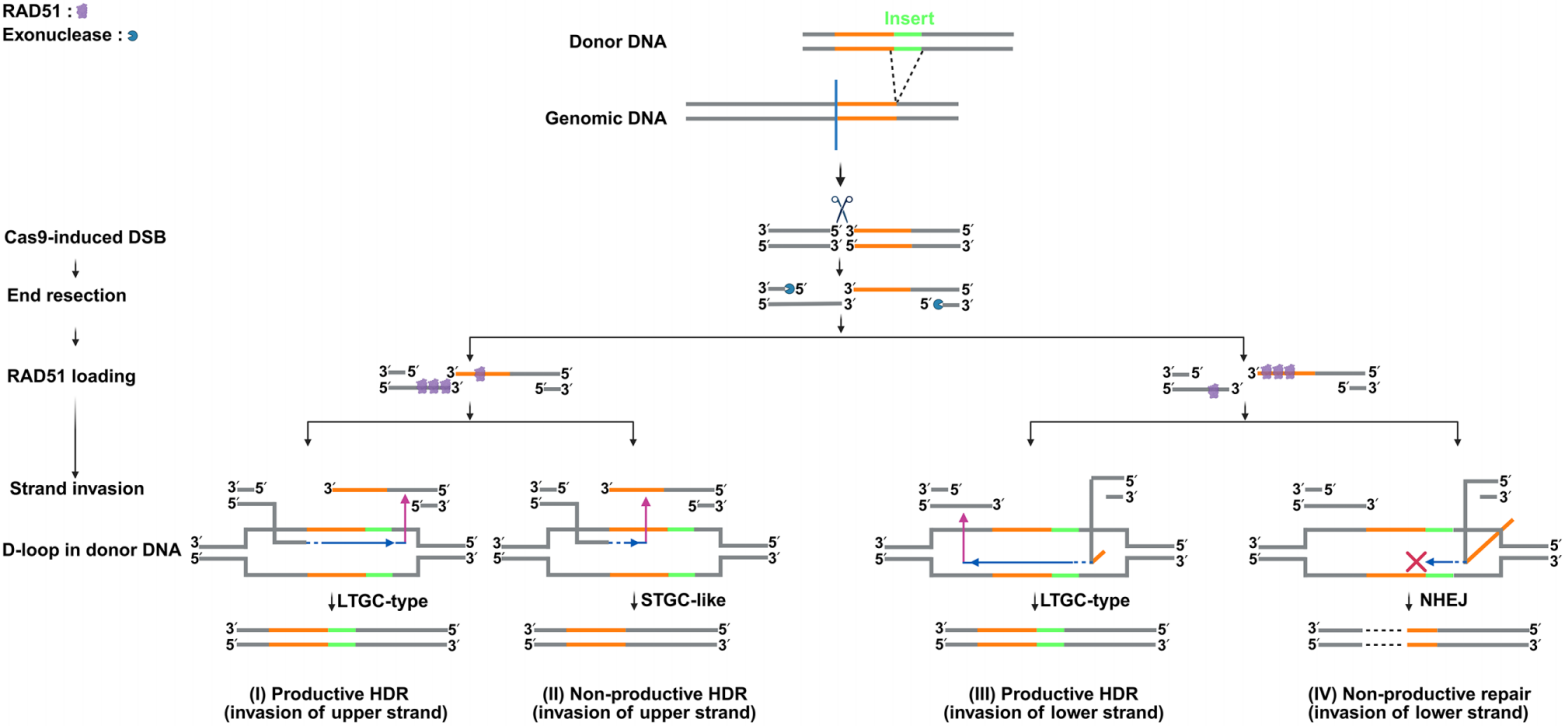
Strand-invasion direction dictates donor integration after a Cas9-induced DSB. The donor insertion site (dashed lines; orange/gray HAs flanking a green insert) is distinct from the Cas9 cut site (blue vertical line). After cleavage, 5′→3′ resection exposes 3′ ssDNA. RAD51 preferentially loads onto one of the two resected 3′ ends and promotes directional strand invasion into the donor, forming a D-loop that primes DNA synthesis. Any apparent asymmetry in RAD51 association could reflect differences in RAD51 loading behavior and differences between the two strands in resection-dependent ssDNA availability (e.g. differences in the extent or kinetics of end resection). DNA polymerase extends the invading 3′ end within the D-loop (blue arrow). Depending on which donor strand is invaded and whether proper homology pairing is achieved, four possible outcomes can occur: (I) Productive HDR (Long-Tract Gene Conversion (LTGC)-type integration)—the invading 3′ end pairs with the upstream HA and synthesizes across the entire insert, generating a long-tract gene conversion. (II) Nonproductive HDR (Short-Tract Gene Conversion (STGC)-like premature annealing)—the 3′ ssDNA invades the upper donor strand, but anneals prematurely to genomic sequence before reaching the upstream HA. This early annealing produces a short conversion tract lacking the insert. (III) Productive HDR (LTGC-type integration)—the 3′ end invades the lower donor strand and initially forms a short, transient unpaired 3′ flap, which is retained during D-loop formation through annealing at an internal homologous segment and subsequently removed by cleavage as a nonhomologous 3′ flap, resulting in a long-tract gene conversion with precise integration. (IV) Nonproductive repair (NHEJ/abortive invasion)—invasion of the lower donor strand produces a long, unpaired 3′ flap when the insert is encountered before homology. This flap leads to abortive strand invasion and NHEJ-mediated end joining without the insert. This schematic integrates established STGC/LTGC concepts with strand-invasion polarity models from previous HDR studies [9–14].

The success of HDR is critically dependent on strand invasion mediated by the recombinase RAD51. After Cas9-induced DSBs, RAD51 assembles on the resected 3′ single-stranded DNA (3′ ssDNA) to form a nucleoprotein filament [9, 10]. The filament invades homologous donor sequence to generate a displacement loop (D-loop) that primes DNA synthesis (Fig. 1). Productive elongation requires effective pairing between the 3′ ssDNA end and a homologous region in the donor that can serve as a template for polymerase extension [11]. If the donor insertion site is misaligned relative to the point of strand invasion, this process may be hindered, reducing HDR efficiency. In practice, sgRNA constraints often prevent the Cas9 cleavage site from precisely overlapping the insertion site [12], leading to positional mismatch between the donor sequence and the strand invasion origin (Fig. 1). As illustrated in Fig. 1, misalignment between the donor template and the strand-invasion origin can channel repair into four mechanistically distinct routes (I–IV; Fig. 1). When the genomic 3′ end invades the upper donor strand, two outcomes are possible: (I) productive HDR, if the invading 3′ end first pairs with the upstream homology arm (HA); or (II) nonproductive HDR with insert loss, if premature annealing occurs before the invasion front reaches the insert [13]. When the genomic 3′ end invades the lower donor strand, two outcomes likewise occur: (III) productive HDR, upon entry into the donor, the invading 3′ end initially encounters the insert and therefore leaves a short, transient unpaired 3′ tail; subsequent pairing at an internal homologous segment establishes the D-loop, during which this short tail is transiently retained and then removed as a nonhomologous 3′ flap, allowing the invasion front to traverse the insert and reach the downstream HA; or (IV) nonproductive repair [insert loss or nonhomologous end joining (NHEJ)], in contrast, when the invading 3′ end carries a much longer unpaired 3′ region, strand invasion is aborted and repair is redirected toward NHEJ-mediated pathways [14]. Although the latter mechanism lacks direct experimental validation, the structural incompatibility implied by the long 3′ flap is theoretically significant. Consequently, the spatial relationship between the donor and strand-invasion origin represents a critical structural determinant governing HDR success or failure.

To mitigate premature annealing caused by such mismatches, a common strategy involves synonymous recoding of donor sequences that are prone to early pairing with the genome [13]. While this method can improve efficiency in specific contexts, its applicability is limited. First, recoding is only relevant when the insertion lies upstream of the RAD51-loaded strand. Second, recoding cannot be applied to conserved regions such as splice sites or protein-coding domains, where even synonymous substitutions may affect expression. Third, its efficacy declines for insertions located >30 bp from the cut site or for donor sequences exceeding 1 kb in length. Importantly, the design space for recoding lacks a predictable mechanistic framework, as the relative positioning of RAD51 and the insertion site is rarely known in advance.

To date, it remains unclear whether strand-invasion polarity constrains donor integration and whether structural design can overcome this limitation. In this study, we used *Aspergillus nidulans* to map directional loading of the RAD51 homolog UvsC [15] at Cas9-induced DSBs and to link invasion direction to donor-integration outcomes. Guided by this insight, we deploy a dual-sgRNA strategy that makes two flanking cuts to create a geometry-matched strand-invasion window, aligning donor HAs with endogenous extension paths. This structural-docking approach facilitates seamless polymerase extension and mitigates nonhomologous sequences that would otherwise trigger premature annealing or abortive strand invasion with a transient unpaired 3′ flap. The strategy releases donor design from positional constraints and yields high integration efficiency across multiple fungal species, underscoring its versatility and broad applicability in genome engineering.

## Materials and methods

### Strains and culture conditions

*Escherichia coli DH5α* was used for plasmid propagation in Luria–Bertani (LB) medium supplemented with 100 μg/ml ampicillin. Fungal strains included *A. nidulans ABPUN, Aspergillus oryzae RIB40 pyrG⁻*, and *Aspergillus fumigatus Af239 pyrG⁻* (genotypes listed in Supplementary Table S1). All strains were maintained on glucose minimal medium (GMM) containing 1% glucose, 10 mM NaNO_3_, 10 mM KH_2_PO_4_, 7 mM KCl, 2 mM MgSO_4_, and 2 ml/l trace elements (pH 6.5). Auxotrophic supplements—0.5 g/l uracil, 0.6 g/l uridine, 0.4 g/l biotin, and 0.4 g/l pyridoxine—were added as required.

### Plasmid construction and donor DNA design

sgRNA target sites were selected using the Benchling CRISPR design tool (https://www.benchling.com), and only those with predicted ontarget scores ≥53 were retained (Supplementary Table S2). To construct the sgRNA expression module, the *Aspergillus flavus* U6 promoter [16], CRISPR RNA (crRNA) scaffold backbone, and U6 terminator were synthesized and cloned into a pUC19 vector. To generate target-specific sgRNA expression constructs, two polymerase chain reaction (PCR) fragments were amplified from the pUC19-U6-crRNA plasmid. The first fragment (U6 promoter + spacer) was amplified using promoter.F (binding the 5′ end of the U6 promoter) and primer R1 (binding the 3′ end of the U6 promoter and containing a 20-bp target-specific spacer). The second fragment (spacer + crRNA scaffold + U6 terminator) was amplified using primer F2 (binding the 5′ end of the crRNA scaffold and containing the same 20-bp spacer) and promoter.R (binding the 3′ end of the U6 terminator). The two PCR fragments were then assembled into the pyrG-marked vector pFC330 via one-step cloning using the Hieff Clone^®^ Universal II Kit (Yeasen, China), yielding functional sgRNA expression plasmids.

Donor DNA templates were designed with ∼1-kb HAs flanking the intended integration site. Donor fragments were PCR-amplified from the genomic DNA of wild-type (WT) *Aspergillus* strains using PrimeSTAR^®^ GXL DNA Polymerase (Takara, Japan). To prevent Cas9-mediated cleavage of the donor, protospacer adjacent motif (PAM) sequences (NGG) were disrupted via synonymous substitutions. When the PAM spanned two codons, only the third nucleotide of the upstream codon was altered to preserve amino acid identity; reverse-strand targets were treated analogously. Final donor constructs were assembled via multifragment fusion PCR. Primer sequences are provided in the Supplementary Table S3.

### Fungal transformation

Conidia were inoculated at a final concentration of 1 × 10^6^ cells/ml into liquid GMM and incubated at 37°C with shaking (220 rpm) for 5–7 h to induce germination. Germlings were collected and incubated in an enzymatic digestion buffer containing 0.8 M NaCl, 9  mM K_2_HPO_4_ (pH 6.0), 3.0  mg/ml yatalase (Takara, Japan), 0.3  mg/ml lyticase (Merck), and 1.6  mg/ml bovine serum albumin at 30 °C with shaking (180 rpm) for 2–3 h to generate protoplasts. Following centrifugation, protoplasts were gently collected and used for polyethylene glycol-mediated transformation with 500  ng of Cas9-sgRNA plasmid and 1  μg of donor DNA. Transformants were plated onto selective GMM and incubated at 37°C for 48–72  h. Genomic integration was confirmed by colony PCR.

Subsequently, genomic integration of the constructs was verified by colony PCR followed by sequencing. Specifically, the DNA fragments amplified using the primer pairs check_F/check_R (for *wA*), trxA_check_F/trxA_check_R (for *trxA-FLAG*), trxA_control_check_F/trxA_control_check_R (for *trxA-FLAG-T_trpc*), napA_check_F/napA_check_R (for *napA–GFP*), napA_control_check_F/napA_control_check_R (for *napA–GFP–T_trpc*), niaD_check_F/niaD_check_R (for *PP-niiA*), niaD_control_check_F/niaD_control_check_R (for *tPP-niiA*), prxA.UP.F/prxA.UP.R (for *prxAC31S,C61S*), Ao.check.F/Ao.check.R (for *A. oryzae wA*), and Af.check.F/Af.check.R (for *A. fumigatus abl1*) were subjected to Sanger (ABI 3730xl DNA Analyzer; Tsingke, China) or Oxford Nanopore Technologies (CWBio, China) sequencing for precise confirmation of the integration event. The positive clone rate was calculated as the percentage of transformants yielding the correct PCR product with the expected sequence out of the total number screened.

### Chromatin immunoprecipitation

Protoplasts transformed with CRISPR/Cas9 constructs were incubated at 30°C for 2–4 h before sampling. Cells were crosslinked with 1% formaldehyde for 15  min at room temperature, followed by quenching with 125 mM glycine for 5  min. Crosslinked samples were washed with ice-cold phosphate-buffered saline and lysed in high-alkaline RIPA buffer (Beyotime, China) supplemented with 1 mM phenylmethylsulfonyl fluoride (PMSF). Nuclei were pelleted by centrifugation, and chromatin was sonicated on ice (30  s on/30  s off, six cycles) to obtain DNA fragments ranging from 200 to 500  bp. The sheared chromatin was incubated overnight at 4°C with anti-FLAG magnetic beads (Sigma–Aldrich, Cat# M8823). After sequential washes with low-salt buffer, high-salt buffer, LiCl buffer, and Tris–EDTA (TE) buffer, immune complexes were eluted and reverse-crosslinked at 65 °C in elution buffer containing 1% sodium dodecyl sulphate (SDS) and proteinase K. DNA was purified using the Magen Gel Extraction Kit (Magen, China) and subjected to quantitative PCR analysis. For each Cas9-induced DSB, symmetrically positioned primer pairs (Supplementary Table S3) were designed on the PAM-proximal and PAM-distal sides at equal distances from the cleavage site. DNA recovered from equal numbers of protoplasts using the same extraction procedure was used as the quantitative PCR (qPCR) template, with equal volumes of each sample added to the reactions. qPCR enrichment signals corresponding to the PAM-proximal and PAM-distal sides were measured for each locus, with the weaker side set to 1 and the opposite side expressed as its relative fold enrichment.

### Microscopy

*NapA–GFP* strains were cultured in 15-mm glass-bottom confocal dishes at a density of 2  ×  10^5^ conidia per well and incubated at 37°C for 7 h to allow hyphal germination. For nuclear visualization, germinated hyphae were stained with Hoechst 33258 (5 μg/ml). Oxidative stress was induced by treatment with 1 mM hydrogen peroxide (H_2_O_2_). Fluorescent mycelia were imaged using a confocal laser scanning microscope (TCS SP8, Leica Microsystems, Germany) equipped with appropriate Leica filter cubes. GFP fluorescence was detected using 488-nm excitation and 510-nm emission settings. Image acquisition and processing were performed using LAS X software (Leica Microsystems).

### Western blotting

Mycelia treated with 0–2 mM H_2_O_2_ for 3  h were flash-frozen in liquid nitrogen and ground to a fine powder. Total protein was extracted using SDS lysis buffer (1% SDS, 10  mM dithiothreitol (DTT)), followed by heating at 98°C for 10 min. The supernatant was collected as the western blot sample. For each sample, 20  μg of protein was mixed with 4× sodium dodecyl sulphate–polyacrylamide gel electrophoresis (SDS–PAGE) loading buffer (Takara, Japan) and loaded per lane. Proteins (20 μg/lane) were separated by SDS–PAGE and transferred to polyvinylidene difluoride (PVDF) membranes in duplicate. Membranes were blocked and incubated overnight at 4°C with mouse monoclonal primary antibodies against FLAG (Thermo Fisher) or GAPDH (Thermo Fisher), each diluted 1:2000 in TBST buffer [25  mM Tris–HCl (pH  7.4), 150  mM NaCl, 0.05% Tween-20]. After three washes (15  min each) in TBST, membranes were incubated for 1  h at room temperature with horseradish peroxidase-conjugated goat anti-mouse IgG secondary antibody (1:5000, Thermo Fisher). Signal was detected using enhanced chemiluminescence reagents (Beyotime, China) and visualized with a Tanon imaging system (Tanon, China).

### RNA extraction and RT-qPCR

Total RNA was extracted using the RNApure Plant Kit (CWBIO, China), and complementary DNA was synthesized with the HiFiScript gDNA Removal RT MasterMix Kit (CWBIO) following the manufacturer’s instructions. Quantitative PCR was performed using the Magic SYBR Mixture (BioTec, China) on a CFX96 Touch™ Real-Time PCR Detection System (Bio-Rad, USA). Gene-specific primers targeting *wA* related to UvsC-loading directionality were used, with *actin A* serving as the internal control (primer sequences, including Q-wA.distal-PAM-F/Q-wA.distal-PAM-R and Q-wA.proximal-PAM-F/Q-wA.proximal-PAM-R, are listed in Supplementary Table S3). Relative gene expression levels were calculated using the 2^−ΔΔ*Ct*^ method and normalized to*actin A*.

### Whole-genome sequencing and off-target analysis

Mycelia of the parental strain (*A. nidulans ABPUN*) and two edited strains (*trxA-FLAG* and *trxA-FLAG-T_trpC*) were collected from liquid cultures and ground in liquid nitrogen. Genomic DNA was extracted using the FastClean Plant Genomic DNA Kit (Cat. No. W0571S, CWBIO, China). Sequencing libraries were constructed following standard Illumina protocols. DNA fragments (∼350 bp) were size-selected with AMPure XP beads (Beckman Coulter, Brea, CA, USA), and libraries were sequenced on an Illumina NovaSeq X Plus platform (Illumina, San Diego, CA, USA) using a PE150 strategy. Raw reads were filtered for quality and mapped to the *A. nidulans* FGSC A4 reference genome (taxid: 227321) using BWA-MEM (v0.7.17). Variants (single-nucleotide polymorphisms (SNPs) and insertions/deletions (InDels)) were identified with GATK (v4.2.6.1) and annotated using SnpEff (v5.1). Potential CRISPR/Cas9 off-target sites were predicted with CRISPRitz (v2.1), considering up to three mismatches to the designed sgRNAs. Variants shared with the WT, with coverage <10×, or with missing genotypes were excluded. Candidate off-target loci were manually inspected with IGV (v2.16.1) to confirm the absence of editing signals. Structural variation (SV) analyses were performed using Manta (v1.6.0) and BreakDancer (v1.4.5), and results were visualized in Circos (v0.69-9). No large-scale SVs or novel copy number variations (CNVs) were detected, confirming genome-wide stability after CRISPR/Cas9 dual-sgRNA editing.

### Statistical analysis

All experiments were performed in triplicate. Chromatin immunoprecipitation followed by quantitative PCR (ChIP-qPCR) data were normalized to input controls using Δ*Ct* values. Statistical comparisons were conducted using one-way ANOVA or two-tailed Student’s *t*-test (GraphPad Prism). A *P*-value of <.05 was considered statistically significant.

## Results

### The RAD51 homolog UvsC exhibits directional loading at Cas9-induced DSBs

**Figure 2.**
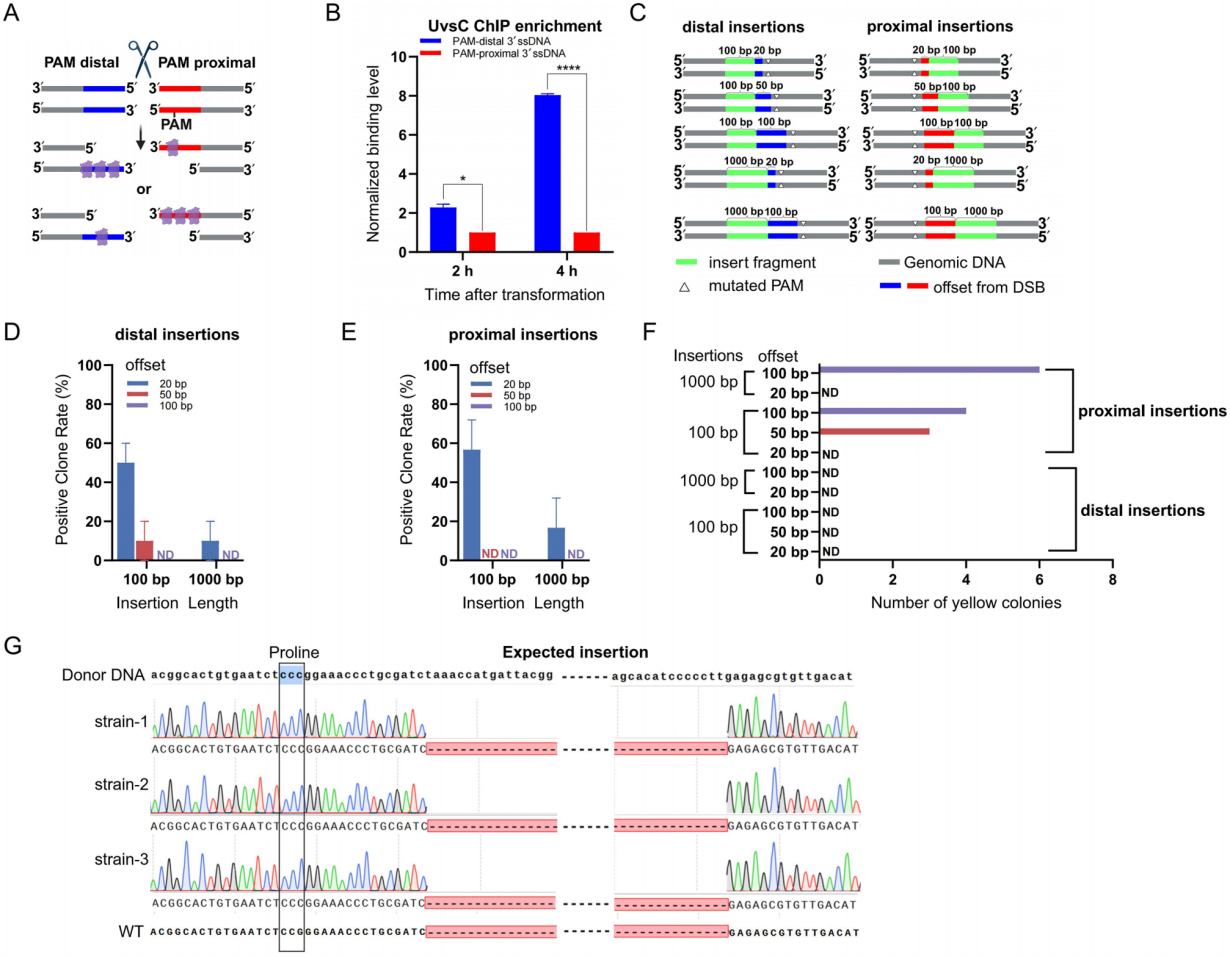
UvsC-loading polarity, insertion-to-DSB distance, and insert length jointly shape HDR efficiency in a single-cut CRISPR design. (**A**) Schematic of asymmetric UvsC loading on the 3′ ssDNA ends exposed by a Cas9-induced DSB at the *wA* locus. A single cut was introduced at position +220. The relative positions of the PAM sites are indicated in the schematic. (**B**) Time course of UvsC ChIP-qPCR enrichment at the two sides (2 and 4 h post-transformation). Primer pairs were symmetrically designed on both sides of the break, with the distal primer placed ∼150 bp from the cut toward the PAM-distal or PAM-proximal direction. Bars show mean ± standard deviation (SD), *n* = 3 independent transformations. Significance by two-tailed *t*-test (*P < .05; ****P < .0001). (**C**) Donor designs. Donor templates contained either a 100-bp or 1-kb insert (green) positioned 20, 50, or 100 bp from the DSB on the distal PAM or proximal PAM side. Each donor was flanked by 1-kb HAs (gray). The PAM sequence on the donor was silently mutated (white triangles) to prevent Cas9 re-cleavage. Distances are measured from the Cas9 cut to the first base of the intended insert. Positive clone rates for distal PAM side (**D**) and proximal PAM side (**E**) insertions. Efficiency was calculated as the percentage of colonies with the correctly inserted fragment. ND, not detected. (**F**) Counts of yellow colonies observed during selection across insertion positions (distal versus proximal PAM side) and distances (20/50/100 bp). Yellow colonies represent donor-templated PAM-site repair without insertion (“PAM-escape” genotype). (**G**) Sanger sequencing of representative yellow colonies (three independent strains) shows donor incorporation lacking the intended insert. In the diagram, the left black box marks the original genomic PAM (mutated in the donor), and the proximal PAM dashed gap indicates the missing insert. All bar plots show mean ± SD from three independent transformations. For each condition, 10 colonies (*n* = 10) were randomly selected from transformation plates for verification.

We investigated whether the RAD51 homolog UvsC exhibits directional loading at Cas9-induced DSBs. As a test locus, we targeted the *wA* gene in *A. nidulans*, a well-characterized pigmentation gene commonly used in fungal genetic studies [16]. A single cleavage site was introduced at position +220 of the *wA* coding region (Fig. 2A). To detect UvsC binding, a FLAG tag was fused to its C-terminus, and the tagged strain was subjected to ChIP-qPCR. Enrichment of UvsC was quantified at the 3′ ssDNA regions flanking the DSB. UvsC showed significantly higher enrichment on the PAM-distal side of the DSB compared to the PAM-proximal (Fig. 2B). At 4 h after transformation, the ChIP signal on the PAM-distal flank was approximately eightfold greater than that on the PAM-proximal. To exclude potential bias caused by unequal primer efficiencies, primer sets were designed on both sides of the Cas9-induced DSB, with distances from the cleavage site of +146 bp, +194 bp, +218 bp, +252 bp, and +644 bp on the PAM-distal and +159 bp, +205 bp, +225 bp, +248 bp, and +666 bp on the PAM-proximal, forming approximate pairs across the two sides. UvsC enrichment was detectable within +252 bp of the cleavage site but not at +644 bp (Supplementary Fig. S1). This loss of signal at longer distances likely reflects fragmentation of extended DNA regions during sonication, confirming that the ChIP-qPCR assay reliably detects protein–DNA interactions within ∼250 bp of the break. Across all primer pairs, the PAM-distal side consistently showed stronger signals than the PAM-proximal side, consistent with the results in Fig. 2B. This asymmetric binding pattern suggests a locus-specific bias in UvsC loading, favoring the PAM-distal side 3′ ssDNA at this Cas9 cleavage site.

To further examine whether the directional loading of UvsC represents a general phenomenon and whether the extent of this bias is consistent across different cleavage sites, we selected five Cas9 cleavage sites distributed along the *wA* coding region, corresponding to AGG, TGG, CGG, and two distinct genomic sites carrying the GGG PAM (Supplementary Fig. S2). Each sgRNA introduced a single cleavage site within the coding region, located +379 bp (AGG), +562 bp (TGG), +220 bp (CGG), +500 bp (first GGG site), and +758 bp (second GGG site) downstream of the ATG start codon, respectively. End-distinguished ChIP-qPCR assays were performed at each site to assess UvsC enrichment on both sides of the DSB. UvsC enrichment was detected at all cleavage sites, confirming the presence of directional loading in each case. However, the extent of asymmetry varied among sites: TGG and CGG displayed a pronounced PAM-distal preference, AGG and GGG showed moderate or negligible bias, whereas GGG-2 exhibited a slight PAM-proximal enrichment (Supplementary Fig. S2). These results indicate that directional loading of UvsC is a general feature of Cas9-induced DSB repair, although the degree of bias differs among cleavage sites. Such spatially biased loading may influence the initial site of strand invasion and may reflect a structural asymmetry in homologous recombination at this locus.

### Integration efficiency depends on UvsC-loading direction and insertion distance

Such spatially biased loading may determine the direction of strand invasion and thereby influence homologous recombination outcomes. To test this, we designed donor integration assays at the previously introduced Cas9 cleavage site located at position +220 of the *wA* coding region (Fig. 2A). We constructed donor templates containing either a 100-bp or 1-kb insertion, positioned at 20, 50, or 100 bp from the Cas9-induced DSB on either side. All donors were flanked by 1-kb HAs, and PAM sequences were silently mutated to prevent re-cleavage (Fig. 2C). The results (Fig. 2D and E; Supplementary Fig. S3A and C showing representative conditions #1–5 for raw plates and PCR validation) showed that when insertions were located on the PAM-distal side of the DSB—corresponding to the UvsC-loading-biased side—the integration efficiency decreased markedly with increasing distance from the cut site. For the 100-bp insert, the positive-clone rate was ∼50% at 20 bp, dropping to ∼20% at 50 bp, and was undetectable at 100 bp. For the 1-kb insert, ∼10% efficiency was observed only at 20 bp, while no positive clones were obtained at longer distances (Fig. 2D). When insertions were placed on the PAM-proximal side of the DSB—opposite the UvsC-loading-biased PAM-distal side—integration efficiencies at 20 bp were comparable to or slightly higher than those on the PAM-distal side. However, with increasing distance, efficiency dropped sharply, indicating that integration on the PAM-proximal side is highly distance-dependent. At 20 bp, the integration efficiencies for the 100-bp and 1-kb inserts were ∼60% and ∼20%, respectively, while no integration was detected at 50 or 100 bp in either case (Fig. 2E; Supplementary Fig. S3B and C showing representative conditions #6–10 for raw plates and PCR validation).

These results demonstrate that regardless of insert length, increasing the distance between the insertion point and the DSB reduces HDR efficiency, and this effect becomes more pronounced at longer distances when the insert lies on the side opposite UvsC loading. This suggests that successful integration requires both alignment between the DSB end and donor HAs and spatial proximity between the HA and the UvsC-loading end. When the insert is located on the UvsC-loading side but at a greater distance from the DSB, strand invasion from the 3′ ssDNA end may not reach the homologous region upstream of the insert. In this case, the invading 3′ end cannot stably pair at the insertion site, and the insert itself obstructs access to the downstream homology segment. As a result, this short region may be looped out of the D-loop, forming a unpaired donor segment. If excessively long, this unpaired segment may hinder polymerase loading and block the initiation of DNA synthesis (IV; Fig. 1). Conversely, when the insert lies on the side opposite the UvsC-loaded end, the 3′ ssDNA may anneal prematurely with the upstream chromosomal sequence before reaching the insert, thereby triggering a repair outcome that excludes the donor insert (II; Fig. 1).

Interestingly, in the experimental groups where the insert was located on the PAM-proximal side of the DSB and ≥50 bp from the cleavage site, a small number of yellow colonies were observed (Fig. 2F and Supplementary Fig. S3B). Sequencing of three randomly selected clones revealed no insert integration, whereas PAM sites had been repaired via donor-templated synonymous mutations (Fig. 2G). This suggests that strand invasion initiated from the RAD51-loaded 3′ end, and the PAM-proximal region of the donor annealed before the insert sequence was engaged, thereby introducing the PAM mutation and escaping further Cas9 cleavage, while the insertion itself was bypassed. This insertionless repair with PAM escape genotype was only observed when inserts were placed on the opposite side of UvsC loading. When inserts were located on the biased PAM-distal side, the 3′ ssDNA first engaged the insertion site, precluding premature annealing, and no yellow colonies were observed (Fig. 2F).

Together, these results indicate that HDR integration efficiency is constrained by spatial compatibility between UvsC-loading direction and donor structure. If the insertion site is not well-aligned with the initiation point of strand invasion, integration may fail due to either unpaired 3′ ends or premature annealing. These discontinuities between the DSB end and donor homology represent a critical bottleneck for conventional nonallelic insertion strategies and call for improved design principles in genome editing.

### Dual-sgRNA strategy aligns donor structure with strand invasion to enhance HDR integration efficiency

**Figure 3.**
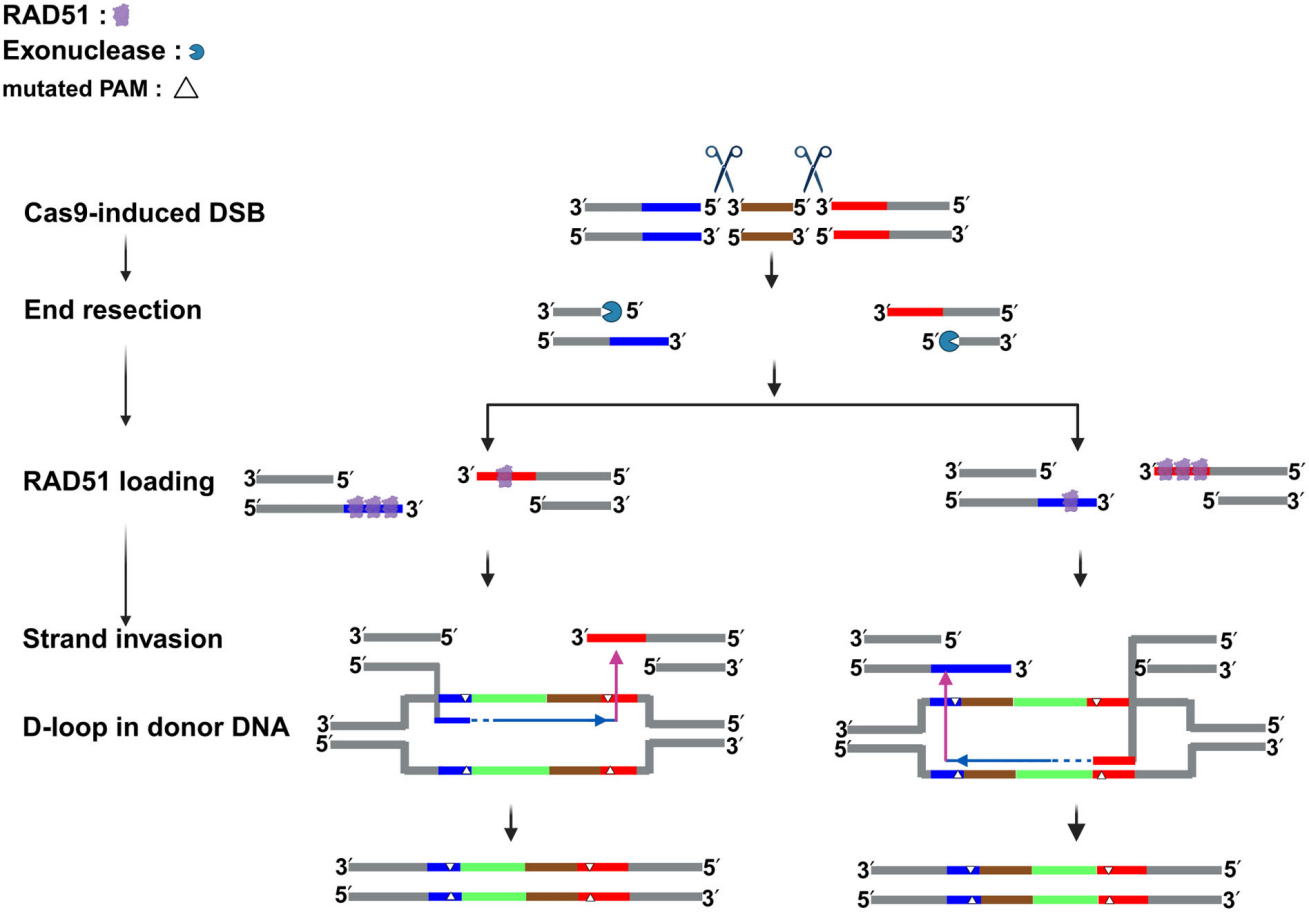
Dual-sgRNA strategy: aligning DSB ends with donor and recipient for enhanced HDR. Two sgRNAs target sites flanking the intended insertion site in the genome, allowing Cas9 to make two precise DSBs and excise a short chromosomal segment. 5′→3′ resection exposes 3′ ssDNA ends that undergo asymmetric RAD51 (UvsC) loading (illustrated by differing RAD51 numbers) and strand invasion into the donor, forming a D-loop that primes DNA synthesis. Any apparent asymmetry in RAD51 association could reflect differences in RAD51 loading behavior and differences between the two strands in resection-dependent ssDNA availability (e.g. differences in the extent or kinetics of end resection). Because the donor insert (green) is colinear with the excised genomic segment and both donor ends are anchored by HAs (red and blue) corresponding to the upstream and downstream cut boundaries, premature annealing and a transiently unpaired 3′ flap configurations are minimized. Donor PAM-disrupting substitutions (white triangles) are included to prevent re-cleavage by Cas9 after integration. The brown segment represents the genomic region excised by the dual-sgRNA cuts, which is reconstituted after donor-mediated repair to restore chromosomal continuity. This geometry improves alignment along the invasion path and increases HDR integration efficiency.

To overcome the integration inefficiencies caused by discontinuity between the DSB end and donor homology, we devised a dual-sgRNA-mediated dual-cut (dual-DSB) insertion strategy (Fig. 3). By introducing two Cas9 cleavage sites flanking the desired insertion site, this approach creates a precisely aligned DSB window, enabling structural compatibility between the donor and the chromosomal break. This design optimizes the alignment between the DSB end and donor HAs, thereby improving HDR efficiency.

**Figure 4.**
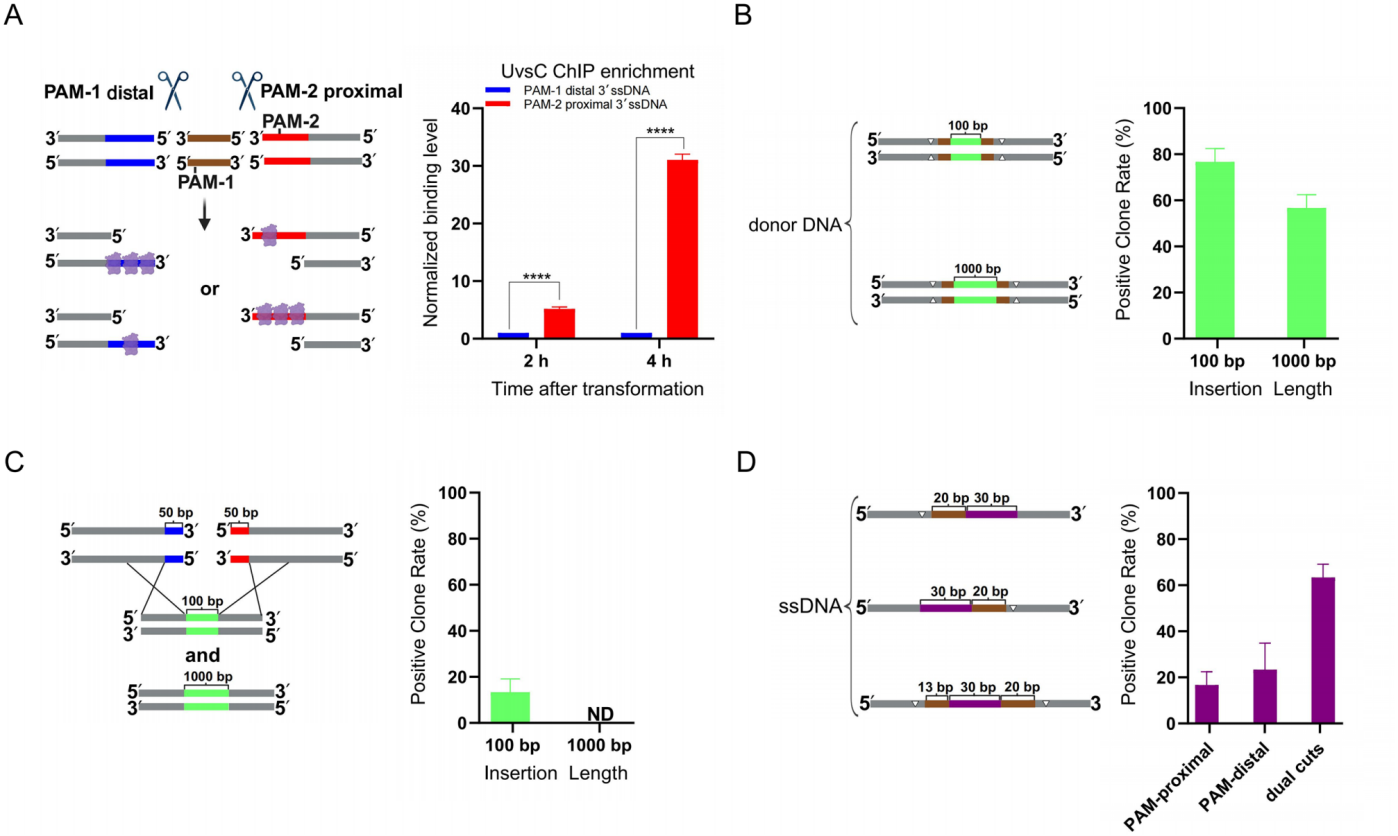
Dual-sgRNA design enhances HDR by matching the donor to a defined DSB window. (**A**) UvsC ChIP enrichment at a DSB window in *wA* defined by two Cas9 cleavage sites (positions +65 and +220 of the *wA* coding region). Primer pairs were symmetrically designed near both cleavage sites, with each distal primer placed ∼120 bp from the cut toward the intervening genomic region. Enrichment on the 3′ ssDNA flanks was measured 2 and 4 h post-transformation (PAM-1, corresponding to the cleavage site at position +220, distal = blue; PAM-2, corresponding to the cleavage site at position +65, proximal = red). Bars, mean ± SD (*n* = 3 independent transformations); two-tailed *t*-test (****P < .0001 for both comparisons). (**B**) Insertion efficiencies for dsDNA donors carrying a 100-bp or 1-kb insert (green) with 1-kb HAs. Donors include PAM-disrupting substitutions (white triangles) to prevent re-cleavage by Cas9. The brown segment marks the genomic region excised by dual-sgRNA cuts and reconstituted during donor-mediated repair. Bar plot shows the percentage of positive clones. (**C**) Donors misaligned relative to the DSB window reduce efficiency. Donors were deliberately misaligned by extending each HA 50 bp beyond the cleavage boundaries, creating an offset between the insert and the genomic window. The bar plot shows the reduced positive-clone rate. ND, not detected. (**D**) Design of ssODN donors with different insertion positions. Each ssODN contains a 30-bp insert flanked by 30-nt HAs. Under the single-cut configuration, the insertion site was placed 20 bp on the PAM-proximal side of the Cas9 cut at position +220 (upper schematic) or 20 bp on the PAM-distal side (middle schematic) within the *wA* coding region. Under the dual-cut configuration, the insertion site was positioned within the dual-DSB window spanning positions +188–+220 (lower schematic). White triangles denote PAM-blocking mutations. Brown segments indicate the physical distance between the insertion site and the Cas9 cut site(s). In the single-cut designs, a single 20-bp brown segment represents the offset from the cut to the insertion site. In the dual-cut design, two brown segments flank the 30-bp insert, measuring 20 and 13 bp, corresponding to the distances from the insertion site to the +220 cut and the second Cas9 cut, respectively; the 13-bp offset is determined by the position of the PAM associated with the second cut. All bar plots show mean ± SD from three independent transformations. For each condition, 10 colonies (*n* = 10) were randomly selected from transformation plates for verification.

Specifically, we introduced two Cas9 cuts at positions +65 and +220 of the *wA* gene to generate a defined dual-DSB system. The donor DNA contained a 1-kb HA on each side of the insert to support precise integration. To determine whether UvsC loading retains its directional bias under dual-DSB conditions, we performed ChIP-qPCR to assess UvsC binding around both DSB sites. At 2 and 4 h after transformation, UvsC enrichment was significantly stronger at the 3′ ssDNA region on the PAM-2-proximal flank of the +65 site (Fig. 4A), suggesting a local structural context more favorable for UvsC loading. Notably, this loading bias was opposite to that observed in the single-DSB system at position +220, where UvsC preferentially bound the 3′ ssDNA region on the PAM-1 distal flank (Fig. 2B). This indicates that the direction of strand invasion under dual-DSB conditions may be influenced by the structural states of both DSB ends. Next, we evaluated the integration performance of 100-bp and 1-kb double-stranded DNA (dsDNA) donors using this dual-cut strategy. Remarkably, the 100-bp donor achieved an integration efficiency of up to 80%, while the 1-kb donor reached 60% (Fig. 4B; Supplementary Fig. S4A and B showing representative conditions #11–12 for raw plates and PCR validation). Compared to single-cut strategies, the dual-sgRNA system significantly improved integration success, especially for long inserts (Fig. 2D and E), by creating a geometrically matched strand invasion window and mitigating donor-to-invasion point mismatches.

To test whether this improvement indeed stemmed from proper alignment between the DSB end and donor homology, we engineered “discontinuity” donor constructs in which the 5′ and 3′ ends of the insert extended beyond the DSB boundaries, creating flaps longer than 50 bp on both sides. This configuration introduced a discontinuity between the DSB end and donor homology and generated unpaired donor regions at both ends. Integration efficiency under these conditions dropped below 10% for both 100-bp and 1-kb donors (Fig. 4C and Supplementary Fig. S5A and C), reinforcing the conclusion that alignment between the DSB end and donor homology is a critical determinant of HDR success.

Given that ssDNA donors are primarily integrated via RAD52-dependent, RAD51-independent pathways (e.g. SSTR or SSA), they may be less sensitive to UvsC-loading direction. To examine whether the dual-cut design also benefits ssDNA donors, we compared their integration efficiency under single- and dual-DSB conditions using a 30-bp insert flanked by 30-bp HAs (Fig. 4D). While integration efficiency for PAM-1-proximal and PAM-1-distal insertion sites under the single-cut system was 20% and 30%, respectively, the dual-cut strategy increased efficiency to 60% (Fig. 4D and Supplementary Fig. S5B and C), suggesting that even in RAD51-independent contexts, spatially precise donor-DSB alignment enhances integration, potentially through more efficient annealing at the DSB ends.

Together, these results demonstrate that the dual-sgRNA strategy improves integration efficiency by precisely removing discontinuity between the Cas9 cut and the insertion site. This design enables complete coverage of the strand-invasion region by the donor, thereby alleviating constraints imposed by UvsC-loading direction. The strategy proves effective for both short and long inserts, and is broadly applicable to both dsDNA and ssDNA donors.

### Validation of the dual-sgRNA strategy for seamless C-terminal tag insertion in target genes

**Figure 5.**
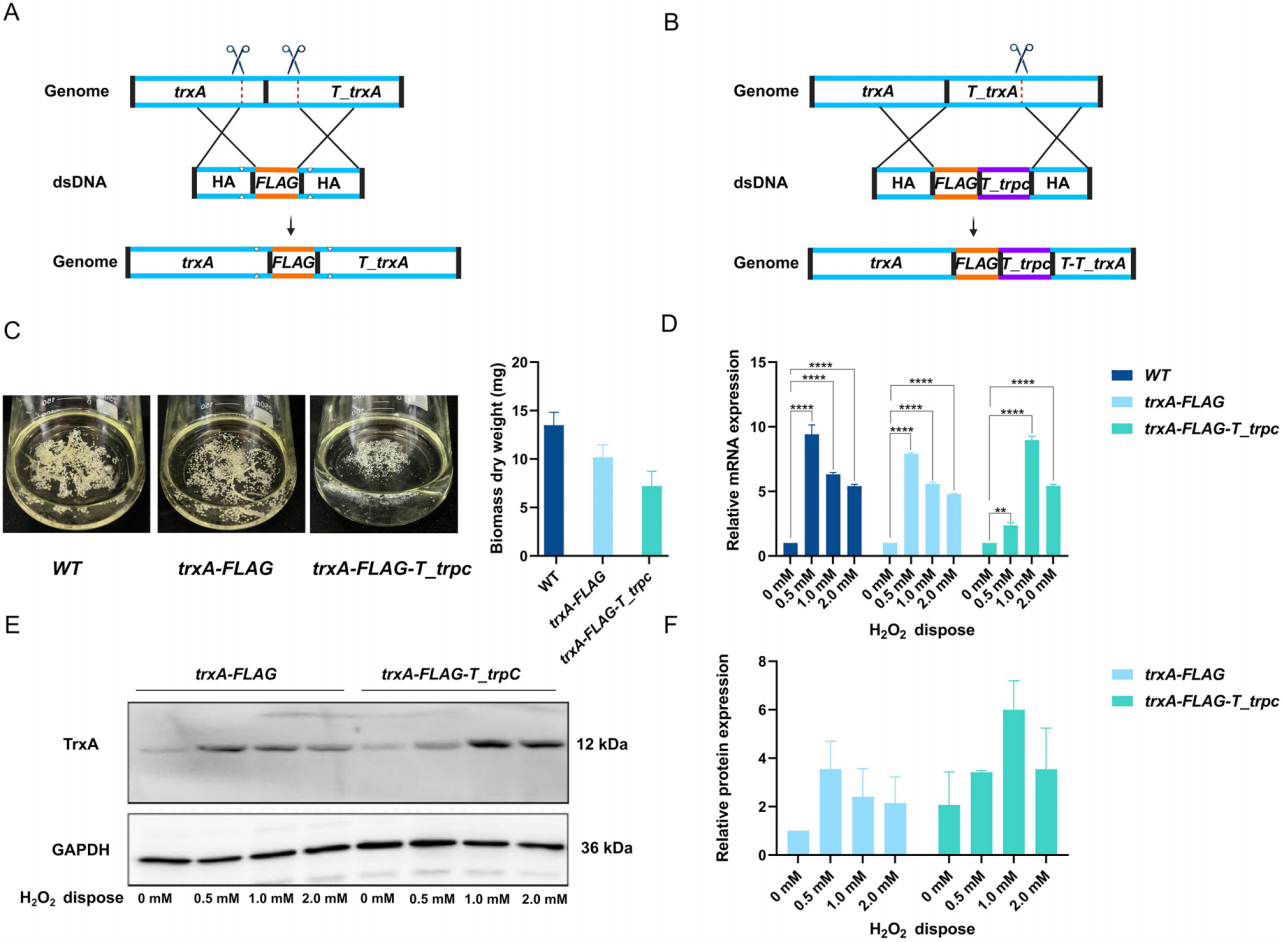
Precise C-terminal FLAG knock-in at the trxA locus using a dual-sgRNA design and functional assessment under H_2_O_2_. (**A**) Dual-sgRNA scheme. Two Cas9 cleavage sites were placed −170-bp upstream and +147-bp downstream of the trxA stop codon to delimit a small genomic window. A dsDNA donor with 1-kb HAs flanking a FLAG tag and an exogenous terminator (T_trpC) replaced the intervening segment by HDR; PAM-disrupting substitutions (white triangles) were introduced to prevent re-cleavage by Cas9. (**B**) Single-sgRNA control. A single cleavage site was positioned +147-bp downstream of the trxA stop codon. The donor carried the FLAG tag, the trpC terminator (T_trpC), and 1-kb HAs, resulting in a truncated fragment of the native T_trxA terminator (T–T_trxA). (**C**) Hyphal growth under oxidative stress. ∼1 × 10^8^ conidia per strain were inoculated into 100 ml GMM containing 1 mM H_2_O_2_ and cultured for 12 h; hyphal growth was photographed. The image shown represents one of three independent experiments. Mycelia were harvested, dried, and weighed to determine dry biomass (mean ± SD, *n* = 3). (**D**) and (**E**) Expression of trxA messenger RNA (mRNA) and TrxA protein in response to H_2_O_2_. After 12 h of growth, mycelia were exposed to 0, 0.5, 1.0, or 2.0 mM H_2_O_2_ for 3 h. qRT-PCR measured trxA mRNA (normalized to GAPDH; mean ± SD, *n* = 3; two-tailed *t*-test; **, **** indicate *P* < .01, .0001). Western blot detected FLAG-tagged TrxA using anti-FLAG; GAPDH was the loading control. (**F**) Quantification of TrxA protein. Band intensities from panel (E) were measured (ImageJ) and normalized to GAPDH; values are mean ± SD (*n* = 3).

To further evaluate the applicability and biological compatibility of the dual-sgRNA strategy for C-terminal tag insertion, we selected *trxA* as a model gene. *trxA* encodes a thioredoxin involved in cellular redox regulation, and its proper expression is critical for maintaining oxidative-stress resistance [17]. Based on the distribution of PAM sites, we designed two sgRNAs targeting sites located −170-bp upstream (cut site 1) and +147-bp downstream (cut site 2) of the *trxA* stop codon (TAG), thereby generating a dual-DSB system with a 320-bp excision window (Fig. 5A). The donor DNA was constructed with 1-kb HAs flanking both sides of the insert and included a *FLAG* epitope tag inserted immediately upstream of the stop codon. To prevent donor cleavage, all PAM sites were silently mutated. This design enabled seamless fusion of the *FLAG* tag without disrupting the native 3′ untranslated region (3′-UTR) or termination signals. Transformants carrying the *trxA-FLAG* fusion were successfully obtained, with an observed integration efficiency of 50%, confirming the robustness and precision of the dual-sgRNA strategy for C-terminal tag insertions (Supplementary Fig. S6A, upper panel, lanes 1–10). For comparison, we constructed a control strain using a conventional single-cut strategy, introducing a single Cas9 cleavage site +147-bp downstream of the *trxA* stop codon. The donor template in this case incorporated both a *FLAG* tag and a strong exogenous terminator (*T_trpC*), resulting in a *trxA-FLAG-T_trpC* fusion construct (Fig. 5B). This design circumvents premature annealing by appending a transcriptional terminator immediately downstream of the tag, achieving an integration efficiency of 30% (Supplementary Fig. S6A, lower panel, lanes 1–10). However, this approach alters the native termination region and poses potential risks to post-transcriptional regulation. Phenotypic and molecular analyses revealed that under 1 mM H_2_O_2_ stress, the growth phenotype of the *trxA-FLAG* strain was comparable to that of the WT, whereas the *trxA-FLAG-T_trpC* strain exhibited reduced oxidative-stress tolerance (Fig. 5C), suggesting that *trxA* transcriptional regulation may have been disrupted. Reverse transcription–qPCR (RT-qPCR) analysis (Fig. 5D) showed that in both *WT* and *trxA-FLAG* strains, TrxA expression was inducible by H_2_O_2_ in a concentration-dependent manner, with expression gradually attenuating at higher concentrations. In contrast, the *trxA-FLAG-T_trpC* strain displayed aberrant expression levels at 0.5 and 1 mM H_2_O_2_. These transcriptional trends were corroborated by western blot results (Fig. 5E and F and Supplementary Fig. S6B), which showed abnormal accumulation of TrxA protein in the *trxA-FLAG-T_trpC* strain. Together, these results suggest that insertion of the exogenous *T_trpC* terminator may perturb the native stress-responsive regulatory dynamics of *trxA*, potentially compromising phenotypic stability. These findings support the dual-sgRNA strategy as a preferred approach for C-terminal tag insertions that preserve endogenous 3′-UTR/termination-dependent regulation, ensuring that the tagged protein retains WT expression and function.

To further evaluate whether the dual-sgRNA design increases off-target risk, we performed whole-genome sequencing and SV analyses to assess genome-wide editing specificity. The chromosomal architecture of all three strains—*WT* (*ABPUN*), *trxA-FLAG*, and *trxA-FLAG-T_trpC*—remained highly conserved, with no detectable large-scale deletions, inversions, translocations, or copy-number variations (Supplementary Fig. S6C). Importantly, no off-target mutations were detected in the dual-cut (*trxA-FLAG*) strain, whereas a single potential off-target site was identified in the single-cut *trxA-FLAG-TtrpC* strain. This site was located in a noncoding region and showed no associated structural or functional gene mutations (Supplementary Table S4). No significant SNP or InDel differences were observed between the edited and WT strains, confirming that the dual-sgRNA CRISPR/Cas9 system does not increase off-target risk or cause genomic instability in *A. nidulans*.

**Figure 6.**
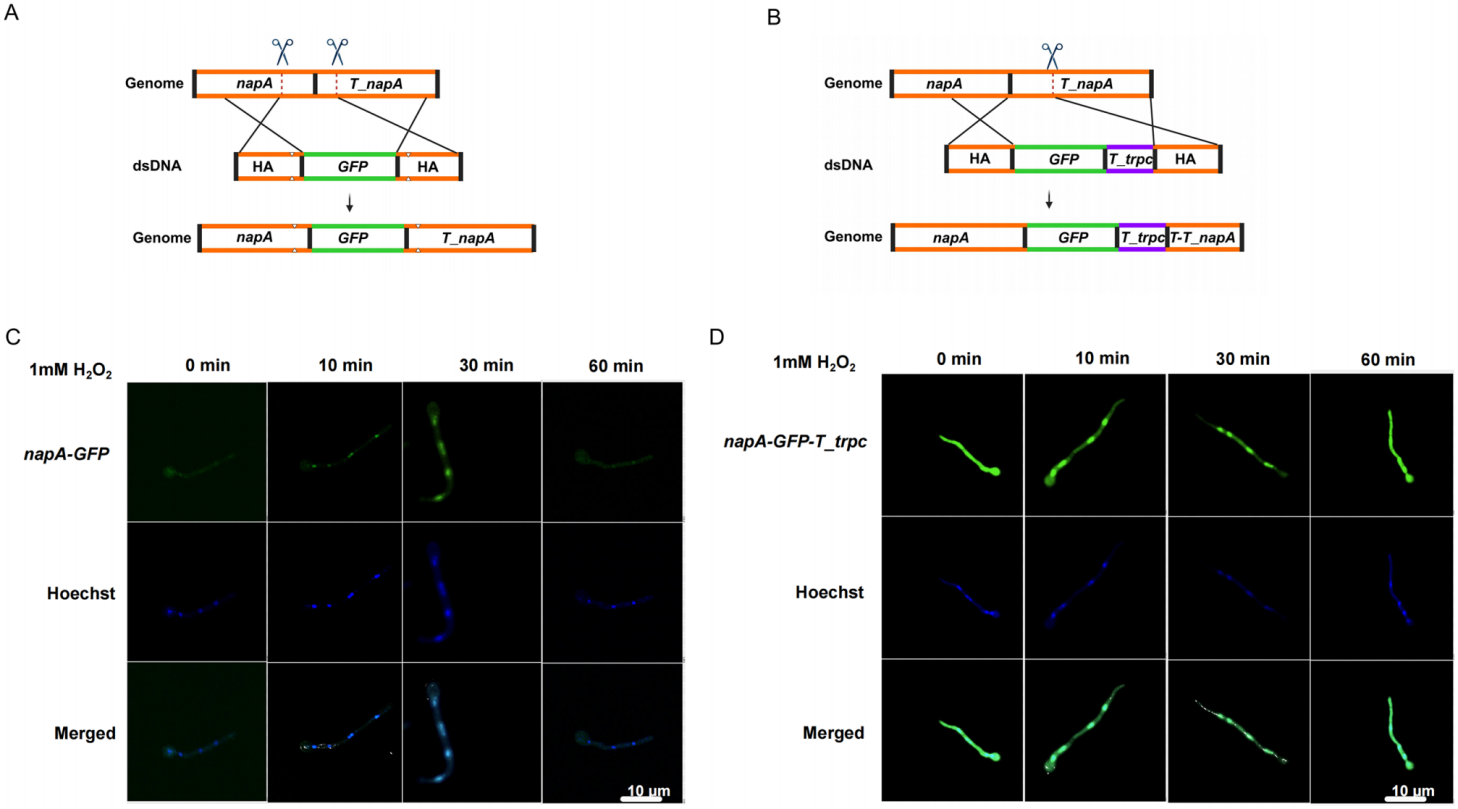
Dual-sgRNA strategy for C-terminal GFP knock-in at *napA* and subcellular localization. (**A**) Dual-sgRNA scheme. Cas9 cleavage sites were placed −260-bp upstream and +58-bp downstream of the *napA* stop codon, defining a 321-bp window. A dsDNA donor with 1-kb HAs flanking *GFP* inserted *GFP* immediately upstream of the stop codon, generating *napA–GFP; napA* terminator (*T_napA)* remained unchanged. PAM-disrupting substitutions were included to prevent re-cleavage. (**B**) Single-sgRNA control. A single cleavage site was positioned 58-bp downstream of the *napA* stop codon. The donor carried *GFP* and *T_trpC* with 1-kb HAs, yielding *napA–GFP–T_trpC*. (**C**) and (**D**) Subcellular localization under oxidative stress. Hyphae were treated with 1 mM H_2_O_2_ for 0, 10, 30, and 60 min and imaged by fluorescence microscopy (GFP, green; Hoechst, blue; merged shown).

To assess whether this strategy is compatible with large epitope tags, we performed C-terminal GFP tagging of NapA, an oxidative-stress-responsive transcription factor [18], thereby enabling subcellular localization analysis under stress conditions. Using the dual-sgRNA strategy, Cas9 cleavage sites were positioned −260-bp upstream and +58-bp downstream of the *napA* stop codon, creating a 321-bp excision window (Fig. 6A). The donor DNA comprised 1-kb HAs flanking the *GFP* coding sequence, which was precisely inserted upstream of the stop codon. All PAM sequences were silently mutated to prevent Cas9 recognition. The resulting *napA_GFP* fusion strain was successfully obtained (Supplementary Fig. S6D). As a control, we employed a single-sgRNA strategy targeting the downstream region and introduced a donor containing *GFP* followed by the *T_trpC* terminator to construct the *napA–GFP–T_trpC* strain (Fig. 6B). Fluorescence microscopy showed that upon 5-min exposure to 1 mM H_2_O_2_, GFP signals in both strains were localized to the nucleus, and returned to the cytoplasm 60 min after stress withdrawal (Fig. 6C and D), consistent with known subcellular dynamics of NapA [17]. Notably, the *napA–GFP–T_trpC* strain exhibited substantially stronger GFP fluorescence than *napA–GFP* (Fig. 6C and D). To substantiate this observation, GFP fluorescence was further quantified in hyphal extracts using a fluorescence spectrophotometer (Supplementary Fig. S6E). The quantitative data agreed with the microscopy results, confirming higher GFP intensity in the *napA–GFP–T_trpc* strain. This observation further supports the notion that exogenous terminators such as *T_trpC* can disrupt native transcriptional regulation and lead to overexpression, introducing potential risks in systems requiring precise expression control. These results again highlight the value of the dual-sgRNA strategy for achieving clean C-terminal integration of large tags without perturbing native gene expression.

### Advantages of the Dual-sgRNA strategy in bidirectional promoter engineering

**Figure 7.**
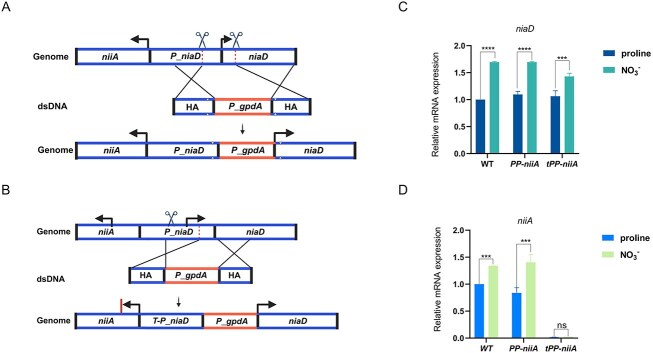
Dual-sgRNA strategy integrates *P_gpdA* immediately upstream of the *niaD* start codon and modulates *niiD*/*niaA* expression. (**A**) Dual-sgRNA scheme. Cas9 cleavage sites were placed −66-bp upstream and +21-bp downstream of the *niaD* start codon (ATG), creating an 87-bp window. A dsDNA donor with 1-kb HAs carrying *P_gpdA* was inserted between *P_niaD* and the *niaD* coding sequence. PAM-disrupting synonymous changes were included to prevent re-cleavage. Paired arrows indicate the direction of transcription. (**B**) Single-sgRNA control. One cleavage site was introduced −66-bp upstream of the *niaD* start codon; a donor carrying *P_gpdA* plus the *niaD* coding sequence was placed downstream of a truncated native promoter (*T-P_niaD*). Paired arrows indicate the direction of transcription. *PP-niiA* denotes the strain carrying a *P_gpdA*–*P_niaD* tandem promoter generated by the Dual-sgRNA strategy. *tPP-niiA* denotes the strain carrying a *P_gpdA*–truncated-*P_niaD* promoter generated by the single-sgRNA strategy. qRT-PCR of *niaD *(**C**) and *niiA *(**D**). Strains were precultured in proline for 8 h, then shifted to nitrate (NO_3_^−^) for 2 h; bars show mean ± SD (*n* = 3 biological replicates). Significance: ns; ***P < .001; ****P < .0001 (two-tailed *t*-test).

Precise modification of promoter regions is a core approach to achieving controllable gene expression. However, when adjacent genes share a bidirectional promoter, conventional replacement strategies often require large-scale reconstruction of regulatory regions, posing significant challenges such as high risk of perturbing nontarget gene expression and increased construct complexity. To address this structural limitation, we evaluated the applicability and structural advantages of the dual-sgRNA strategy in editing complex regulatory elements. As a model, we selected the functionally complementary genes *niaD* and *niiA* in *A. nidulans*, which encode nitrate reductase and nitrite reductase, respectively, and share an ∼1.2-kb upstream intergenic region functioning as a native bidirectional promoter (Fig. 7A). In the dual-sgRNA strategy, we designed two Cas9 cleavage sites located −66-bp upstream and +21-bp downstream of the *niaD* start codon, thereby defining a precise DSB window around the target region. The donor consisted of a 1-kb segment of the *niaD* promoter (*P_niaD*), the strong constitutive promoter *P_gpdA*, and a 1-kb *niaD* coding sequence; all relevant PAM sites within the donor were silently mutated to prevent Cas9 re-cleavage (Fig. 7A). Within this window, we assembled two cassette configurations at the *niiA* locus (Fig. 7B): *PP-niiA*, denoting *niiA::[P_niaD-P_gpdA-niaD*] with an intact *niiA*-facing *P*_*niaD*; and *tPP*-*niiA*, denoting *niiA*::[*tP*_*niaD-P*_*gpdA-niaD*] in which *P*_*niaD* is truncated on the *niiA*-facing side, partially disrupting the native *niiA* promoter. Successful construction of both genotypes was confirmed by colony PCR (Supplementary Fig. S7A). Consistent with the designs, growth assays showed that all strains grew comparably on proline, whereas on nitrate (NO_3_^−^) only *PP*-*niiA* retained WT growth, while *tPP*-*niiA* exhibited marked growth impairment (Supplementary Fig. S7B and C).

qRT-PCR analysis confirmed that in both engineered strains, *niaD* expression levels were substantially higher than in the WT and were no longer inducible by nitrate (Fig. 7C), indicating that the constitutive promoter *P_gpdA* drove *niaD* transcription. Notably, *PP-niiA* retained WT levels of *niiA* expression and preserved its nitrate-inducible response, whereas *tPP-niiA* showed a strong reduction of *niiA* expression and a complete loss of inducibility (Fig. 7D), consistent with partial disruption of the native *niiA* promoter in the *tPP-niiA* configuration. These results highlight the dual-sgRNA strategy as a structurally precise and functionally compatible approach for promoter engineering, particularly in densely packed regulatory contexts such as bidirectional promoters.

### Dual-sgRNA with a dsDNA donor enables efficient long-range point-mutation editing

In genome editing, single-stranded oligodeoxynucleotides (ssODNs) are widely used in combination with a single Cas9-induced DSB to introduce single-nucleotide or short-range substitutions due to their simplicity and efficiency [8]. However, when the distance between two target sites exceeds the typical Cas9 repair range (approximately ±30 bp) [8], simultaneous replacement becomes inefficient. This limitation likewise arises from an “annealing precedence” effect during strand invasion, where the donor preferentially aligns with sequences proximal to the cut site, leaving distal variants unincorporated.

**Figure 8.**
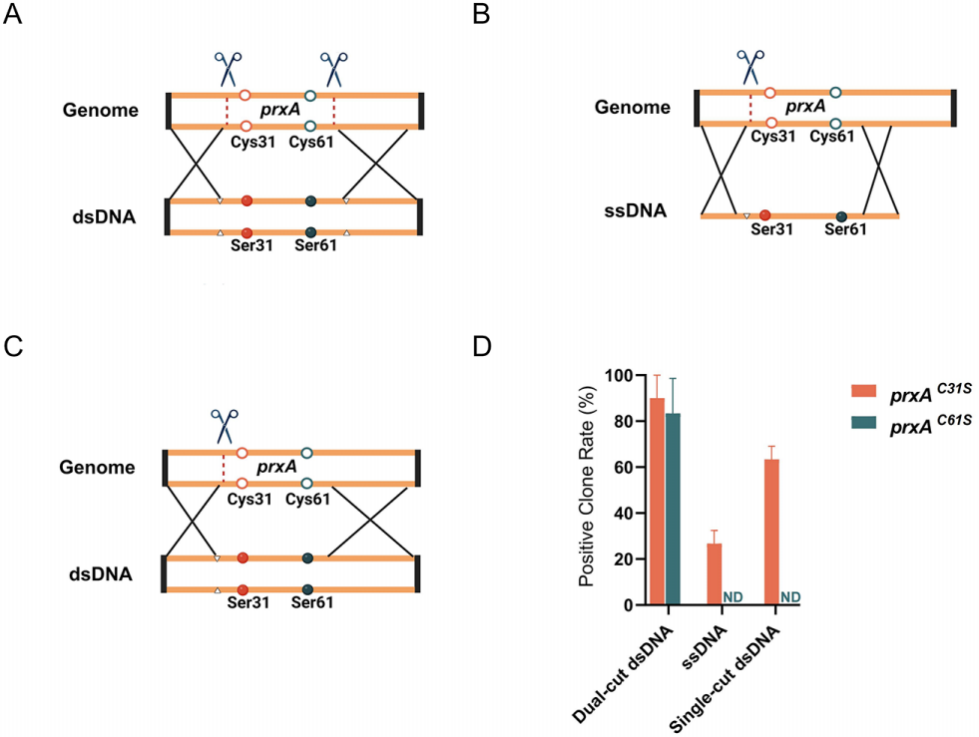
Dual-sgRNA-mediated double-point mutagenesis at the prxA locus using dsDNA or ssODN donors. (**A**) Dual-sgRNA scheme. Cas9 cleavage sites were placed +130-bp upstream and +266-bp downstream of the prxA start codon (ATG), defining a 396-bp window. A dsDNA donor carrying 1-kb HA and two missense substitutions (Cys31Ser and Cys61Ser; orange symbols denote the 31st-position mutation, green symbols denote the 61st-position mutation, hollow circles represent WT Cys, and filled circles represent mutant Ser) in the central region was used for HDR. PAM-disrupting substitutions (white triangles) were included to prevent re-cleavage by Cas9. (**B**) Single-sgRNA with ssODN donor. A single cleavage site was positioned +130-bp downstream of the start codon. An ssODN donor encoding Cys31Ser and Cys61Ser, flanked by 30-nt HAs, was supplied. (**C**) Single-sgRNA with dsDNA donor. A single cleavage site was introduced +130-bp downstream of the start codon. A dsDNA donor carrying the Cys31Ser and Cys61Ser mutations and flanked by 1000-nt HAs was co-delivered. (**D**) Editing outcomes at the two sites (Cys31Ser and Cys61Ser) using dsDNA or ssODN donors. Bars show the percentage of positive clones (mean ± SD, *n* = 3); ND, not detected.

To evaluate the applicability of the dual-sgRNA strategy for long-range point mutation editing, we selected *prxA*, a redox-related gene in *A. nidulans*, as a model. *prxA* encodes a peroxiredoxin involved in oxidative-stress response. Two catalytically essential cysteine residues—Cys31 and Cys61—were chosen as targets for spatially separated point mutations. Two Cas9 cleavage sites were designed at +130- and +266-bp downstream of the start codon, forming a 136-bp editing window (Fig. 8A). Cys31 and Cys61 are located 29-bp downstream of the first cut and 22-bp upstream of the second, respectively, with a 92-bp intersite distance. A dsDNA donor was constructed with 1-kb HAs flanking both sides and incorporated TGC→AGC substitutions at both positions, thereby converting Cys31 and Cys61 to serine. Synonymous mutations were introduced at PAM sequences within the donor to prevent re-cleavage by Cas9.

Experimental results showed that under the dual-cut configuration, 100% of the positive clones carried both substitutions (Fig. 8D and Supplementary Fig. S8), demonstrating high efficiency and fidelity of dual-site editing across a long range. As a control, we tested an ssODN donor harboring the same substitutions, with a 30-bp HA upstream of cut site 1, together with a PAM-blocking mutation (Fig. 8B and Supplementary Fig. S9). Under this single-cut configuration, the editing efficiency at Cys31 was 30% (Supplementary Fig. S9), while no mutation was detected at Cys61 (Fig. 8C), indicating severe limitations in efficiency and range using the ssODN-based strategy. To further compare cutting configurations, we performed a single-cut dsDNA donor experiment using the first cleavage site of the dual-cut design (Fig. 8C and Supplementary Fig. S10). This configuration yielded markedly reduced editing frequencies compared with the dual-cut dsDNA strategy, confirming that simultaneous dual cleavage is critical for efficient long-range replacement. Functional assays revealed that the mutant strain exhibited heightened sensitivity to H_2_O_2_, similar to the *prxA* knockout, indicating complete loss of enzymatic activity due to the dual substitution (Supplementary Fig. S11). Together, these findings demonstrate that the dual-sgRNA strategy, when paired with a dsDNA donor, effectively overcomes spatial limitations and enables high-fidelity, coordinated replacement of distant point mutations. This approach significantly outperforms conventional ssODN-based editing and provides a powerful tool for multisite precision engineering and complex genomic modifications.

### Validation of the dual-cut strategy across *Aspergillus* species

To evaluate the cross-species applicability of the dual-sgRNA-mediated dual-cut strategy in filamentous fungi, we selected *wA*, a pigment-related gene in *A. oryzae* [16], and *abl1* in *A. fumigatus* [19] as model loci for assessing gene-insertion efficiency. For each locus, two Cas9 cleavage sites were designed upstream and downstream of the insertion point to generate a dual-DSB window, with the insertion fragment positioned ∼50-bp downstream of the first cut site. Both single- and dual-sgRNA strategies were tested using donors of different lengths. All donors were dsDNA constructs containing 1-kb HAs flanking either a 100-bp or 1-kb insert, with synonymous mutations introduced at PAM sites to prevent re-cleavage by Cas9. Representative transformation plates in *A. oryzae* showed visibly higher colony numbers under the dual-sgRNA configuration than under the single-sgRNA configuration for both 100-bp and 1-kb donors (Supplementary Fig. S12A). A similar pattern was observed in *A. fumigatus*, where dual-sgRNA yielded more colonies than single-sgRNA for both donor lengths (Supplementary Fig. S12B).

**Figure 9.**
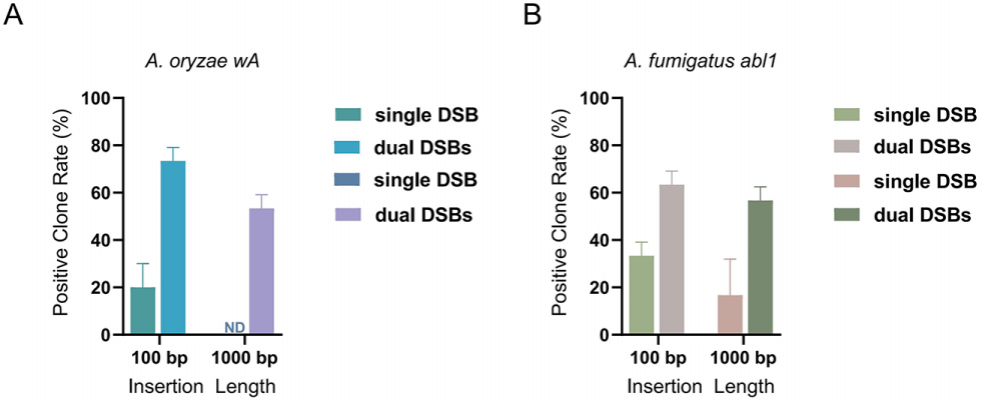
Dual-sgRNA design improves integration across insert lengths in *A. oryzae* and *A. fumigatus*. (**A**) *A. oryzae* wA locus. Cas9 cleavage sites were placed +58- and +174-bp downstream of the start codon (ATG). A dsDNA donor with 1-kb HA carried an insert of 100 bp or 1 kb, positioned ∼50-bp downstream of the cleavage site; PAM-disrupting synonymous changes were included to prevent re-cleavage. The bar chart shows positive clone rates under single-DSB versus dual-DSBs for both insert lengths (mean ± SD, *n* = 3). (**B**) *A. fumigatus* abl1 locus. Cleavage sites were placed + 20- and +199-bp downstream of the start codon (ATG). Donor design and insert positioning were identical to panel (A). The bar chart summarizes positive clone rates under single-DSB versus dual-DSBs for 100-bp and 1-kb inserts (mean ± SD, *n* = 3). All bar plots show mean ± SD from three independent transformations. For each condition, 10 colonies (*n* = 10) were randomly selected from transformation plates and confirmed by PCR or Sanger sequencing.

In *A. oryzae*, the two *wA* cleavage sites were located +58- and +174-bp downstream of the start codon, respectively. Under the single-cut configuration, integration efficiencies were 20% for the 100-bp insert and 0% for the 1-kb insert, with no positive colonies detected for the latter. In contrast, the dual-cut strategy significantly increased integration efficiency to 80% and 60%, respectively (Fig. 9A andSupplementary Fig. S12C). Similarly, for *A. fumigatus*, the *abl1* target sites were positioned +20- and +199-bp downstream of the start codon. The single-cut approach yielded 30% and 20% integration efficiencies for the 100-bp and 1-kb fragments, respectively, while the dual-cut approach increased these to 60% and 50% (Fig. 9B and Supplementary Fig. S12C). These results demonstrate that the dual-sgRNA-mediated dual-cut strategy substantially enhances insertion efficiency in diverse filamentous fungal species. Notably, the improvement is more pronounced for medium-to-long DNA fragments. Compared with conventional single-cut methods, this strategy offers higher positive-clone rates and robust cross-species applicability, underscoring its broad potential for genome engineering in filamentous fungi.

## Discussion

This study systematically establishes and validates that spatial coordination between donor configuration and strand-invasion trajectory as a critical determinant of integration efficiency in CRISPR-HDR. We show that RAD51 (UvsC in *A. nidulans*) loads directionally at Cas9-induced DSBs, initiating strand invasion from the 3′ ssDNA flap. When the insertion site is misaligned—either upstream or downstream—from this 3′ entry point, repair is prone to failure due to structural “dangling” or “premature annealing,” particularly compromising long inserts. To overcome this constraint, we developed a dual-sgRNA strategy that introduces flanking cuts precisely around the intended insertion site, generating a bidirectional strand-invasion window aligned with both HAs of the donor. This structural alignment enables seamless docking to the chromosomal DNA extension paths, markedly improving insertion efficiency and expression fidelity across multiple loci, fragment sizes, and fungal models.

Several strategies have been explored to enhance HDR efficiency in CRISPR-based genome editing [20–22]. For instance, inhibition of key components in the NHEJ pathway, such as DNA-PKcs and Lig4, has been shown to suppress competing repair mechanisms and favor HDR outcomes [23–25]. Alternatively, small-molecule enhancers like RS-1 and Scr7 have been used to pharmacologically bias cells toward HDR [26, 27], while overexpression of recombination factors such as RAD51 has improved strand invasion and D-loop formation rates [28–30]. However, these approaches primarily modulate the global repair environment and do not directly address the geometric compatibility between donor configuration and strand-invasion direction during HDR. Beyond these biochemical manipulations, researchers have also explored various structure-oriented editing strategies to improve Cas9 editing outcomes. Among them, dual-sgRNA systems have been applied in diverse biological models—including mice, *Caenorhabditis elegans*, and fungi—to enhance cutting precision or facilitate large-fragment deletions [31–35]. These studies demonstrated that dual-cut systems can significantly enhance genome-editing efficiency, yet the mechanistic basis for this improvement remains unclear, and how such designs could be structurally optimized to further improve HDR precision has not been explored. This mechanistic gap directly limits HDR performance in complex insertion tasks—such as epitope tagging or promoter replacement—spatial misalignment between the donor and chromosomal strands can hinder D-loop stability and severely compromise integration efficiency [20, 36]. To address this issue, we introduce a structural-docking model for HDR, in which dual-cut donor alignment is achieved through precisely positioned flanking DSBs that generate a bidirectional strand-invasion window. This design enables both HAs of the donor to align with the endogenous DNA synthesis directions, thus achieving spatial congruence and significantly improving both integration rates and expression fidelity. Mechanistically, this model is consistent with the “cut-to-mutation distance effect” described by Paquet *et al.* [37], which demonstrated that HDR efficiency is inversely related to the distance between the Cas9 cleavage site and the desired mutation. Our strategy extends this principle by structurally redefining the optimal integration site, offering a geometric framework for improving HDR precision and performance.

Building upon the mechanistic foundation of the “structural-docking” model in enhancing HDR efficiency, we further explored its applicability and advantages across diverse editing scenarios. This strategy is particularly advantageous in precision insertion tasks—such as epitope tagging (Fig. 5), promoter replacement (Fig. 7), or dual-site mutagenesis (Fig. 8)—where functional elements flanking the donor are structurally constrained and cannot be altered. In such contexts, precise alignment between the DSB end and donor HAs relative to the chromosomal strand-invasion direction becomes critical. Misalignment due to donor polarity mismatch, end displacement, or premature annealing can disrupt D-loop formation or stall DNA synthesis, substantially lowering integration efficiency. While recoding strategies can sometimes mitigate this by minimizing the distance between cut and insertion site, they often fail in longer, tightly packed, or uneditable sequences due to donor distortion or orientation conflict [37, 38]. In contrast, our dual-cut design introduces a coordinated strand-invasion window flanking the insertion site, enabling both donor arms to seamlessly align with the chromosomal synthesis direction without altering the native sequence context. This configuration consistently yielded enhanced integration efficiency across multiple fungal species and construct types, offering strong modularity and cross-system compatibility. It thus provides a structurally standardized framework for high-fidelity HDR in complex genome engineering tasks.

Previous studies have shown that Cas9 tends to remain bound to the PAM-proximal side after cleavage, creating transient asymmetry at DNA ends and influencing repair-pathway choice [39–41]. Although these studies did not directly examine recombinase recruitment, such asymmetric retention raises the possibility that it could also affect where RAD51 is loaded during repair. To test this hypothesis, we analyzed RAD51 (UvsC) binding in *Aspergillus*. As shown in (Fig. 2A and B) and (Supplementary Fig. S2), UvsC exhibited directional enrichment at Cas9-induced DSBs, but the direction and magnitude of asymmetry varied among loci: TGG and CGG sites showed clear PAM-distal enrichment, AGG and GGG displayed little or no bias, whereas GGG-2 even showed mild PAM-proximal preference. These results indicate that the polarity of RAD51 loading is not determined by Cas9 binding orientation but is more likely governed by local sequence context and chromatin environment. It should be noted that previous studies of Cas9 retention effects were mainly based on mammalian *in vitro* experiments [39–41], which captured early, transient repair events and may only reflect temporary changes in DNA-end accessibility or pathway selection, rather than the dynamic behavior of Cas9 or recombinase loading within the cellular chromatin environment. In contrast, our ChIP-qPCR assays capture recombinase binding under steady-state chromatin conditions, where rapid end resection, replication protein A (RPA) coating, and mediator-assisted RAD51 assembly likely mask such transient asymmetry. Furthermore, differences in chromatin structure and DNA-repair kinetics between filamentous fungi and mammalian cells may explain why stable, retentiondriven polarity was not observed in our fungal system.

While our study underscores the critical role of geometric alignment between donor configuration and strand-invasion direction in enhancing HDR efficiency, several mechanistic questions remain. First, the precise determinants governing the directional loading of RAD51 at Cas9-induced DSBs remain incompletely defined. Emerging evidence suggests that factors such as local DNA sequence [42, 43], chromatin accessibility [44], and DSB-end structure can influence RAD51 recruitment and polarization [28, 45, 46], yet a unified model is lacking [28, 47]. Second, the strand invasion and D-loop formation processes remain experimentally opaque due to the absence of real-time visualization tools. Future integration of CRISPR-based live-cell imaging platforms or high-resolution chromatin tracking will be crucial to directly observe these dynamic intermediates [48–50]. Third, although our dual-sgRNA strategy shows consistent efficiency across multiple fungal species, its performance and biosafety in more complex systems—such as human pluripotent stem cells, higher plants, or germline contexts—require systematic investigation. Despite ongoing efforts to engineer directional nucleases or to modulate recombinase loading to enhance HDR [51], our findings reveal a fundamental geometric limitation of single-cut designs. Specifically, strand invasion and donor integration are often compromised due by misalignment between the DSB architecture and the repair template; such designs inherently lack the spatial configuration needed to achieve dual-arm alignment with the chromosomal DNA strands. In contrast, the dual-cut structural-docking strategy enables spatially coordinated strand invasion and robust donor engagement, providing a general design principle for precise, HDR-mediated genome editing.

## Supplementary Material

gkag095_Supplemental_Files

## Data Availability

The whole-genome sequencing data from this study have been deposited in the NCBI Sequence Read Archive (SRA) under the BioProject accession number PRJNA1358014. The individual BioSample accessions are SAMN53096348 (ABPUN), SAMN53096349 (trxA-FLAG), and SAMN53096350 (trxA-FLAG-T_trpC). The corresponding SRA run accessions are SRR35963148, SRR35963147, and SRR35963146. These data are publicly available at https://www.ncbi.nlm.nih.gov/sra.
