# Supplementary material for: Dual-single-guide RNA strategy improves CRISPR-mediated homology-directed repair in Aspergillus
Source: Nucleic Acids Res. 2026 Feb 5;54(4):gkag095. doi: 10.1093/nar/gkag095 (PMC12873602; doi:10.1093/nar/gkag095)
Supplement: gkag095_Supplemental_Files [file gkag095_supplemental_files.zip › Supplementary-Figures-revision2.pdf]

Supplementary Material

Dual-sgRNA Strategy Enhances Homology-Directed Repair Efficiency in CRISPR-Mediated Genome Editing

Mingxin Fu[1], Jing Wang[1], Jingyi Li[1], Yao Zhou[1], Xiaofei Huang[1], Zehan Jia[1], Yiqing Luo[1],Xinyu Tan[1],Yan Gao[1],Bingzi Yu[1],Yuting Duan[1],Qianyun Bu[1],Xiaoying Li[1],Yifan Wang[1],Naoki Takaya[3], Shengmin Zhou[1,2,*]

[1] State Key Laboratory of Bioreactor Engineering, School of Biotechnology, East China University of Science and Technology, Shanghai, 200237, P R China.

[2] State Key Laboratory of Natural and Biomimetic Drugs, Peking University, Beijing, 100191, P R China.

[3] Faculty of Life and Environmental Sciences, Microbiology Research Center for Sustainability, Tsukuba Institute for Advanced Research, University of Tsukuba, Tsukuba, Ibaraki 305-8572, Japan.

[*] Correspondence:zhoushengmin@ecust.edu.cn

Supplementary Figure S1. Detection boundary of UvsC occupancy determined by ChIP–qPCR.

Supplementary Figure S2. Directional loading of UvsC at Cas9 cleavage sites located at different positions in the *wA* locus.

Supplementary Figure S3. Validation of distance- and insert-length-dependent donor integration at the *wA* locus (Cas9 cut at +220 bp).

Supplementary Figure S4. Length-dependent donor integration in the dual-DSB system at the *wA* locus (Cas9 cuts at +65 bp and +220 bp).

Supplementary Figure S5. Donor-mediated integration at the *wA* locus of *A. nidulans*.

Supplementary Figure S6. Validation of dual-sgRNA-mediated C-terminal tagging at the *trxA* and *napA* loci.

Supplementary Figure S7. Validation of donor integration at the *niiA* locus using dual- and single-sgRNA strategies.

Supplementary Figure S8. Validation of *prxA* double-point mutagenesis using a double-stranded DNA donor.

Supplementary Figure S9. Validation of *prxA* point-mutation editing using an ssODN donor.

Supplementary Figure S10. Validation of *prxA* double-site editing using a single-cut CRISPR design and a double-stranded DNA (dsDNA) donor.

Supplementary Figure S11. Growth assay of *prxA* point-mutation strains under hydrogen peroxide stress.

Supplementary Figure S12. Donor integration at the *wA* locus of *A. oryzae* and the *abl1* locus of *A. fumigatus* under single- and dual-sgRNA configurations.

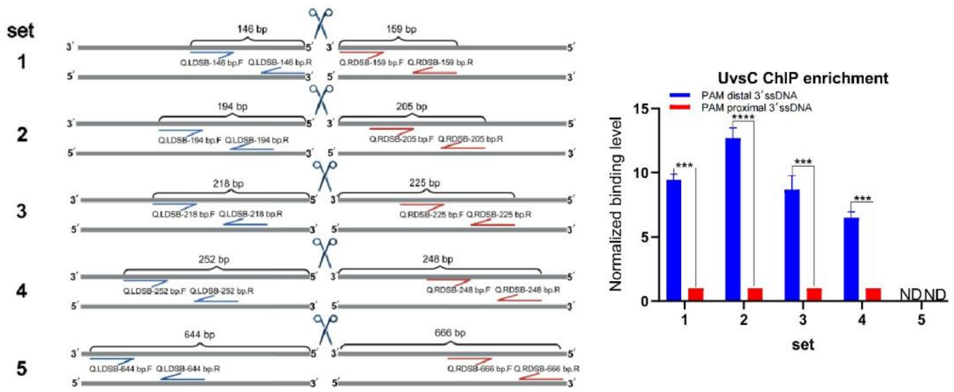

Supplementary Fig. S1. Detection boundary of UvsC occupancy determined by ChIP–qPCR. To determine the detectable range of UvsC around a Cas9-induced DSB (cut site at +220 bp), five PCR amplicons were designed flanking the break. For each amplicon, the indicated distance denotes the genomic distance between the DSB and the end of the amplicon farthest from the break. Amplicons located on the PAM-distal side are shown in blue, and those on the PAM-proximal side are shown in red. The right panel presents the corresponding ChIP–qPCR results, with blue bars representing PAM-distal amplicons and red bars representing PAM-proximal amplicons. “ND” denotes no detectable enrichment. Data represent three independent experiments. ***$P < 0.001$; ****$P < 0.0001$.

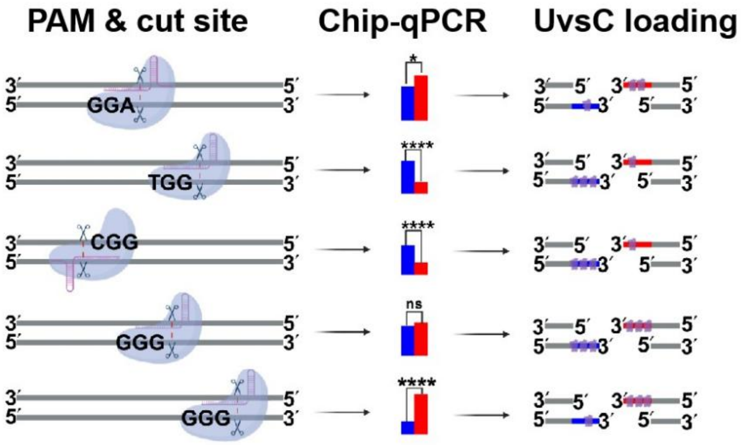

Supplementary Fig. S2. Directional loading of UvsC at Cas9 cleavage sites located at different positions in the *wA* locus. Five Cas9 cleavage sites positioned at different genomic locations in the *wA* locus were examined by ChIP–qPCR distinguishing the PAM-proximal and PAM-distal sides of each break. Their positions relative to the ATG start codon are: +379 bp, +562 bp, +220 bp, +500 bp and +758 bp.The middle panel shows UvsC enrichment on the PAM-proximal (red) and PAM-distal (blue) sides, and the right panel illustrates the inferred direction of UvsC loading. Data represent three independent experiments; ns, not significant; *P < 0.05; ****P < 0.0001.

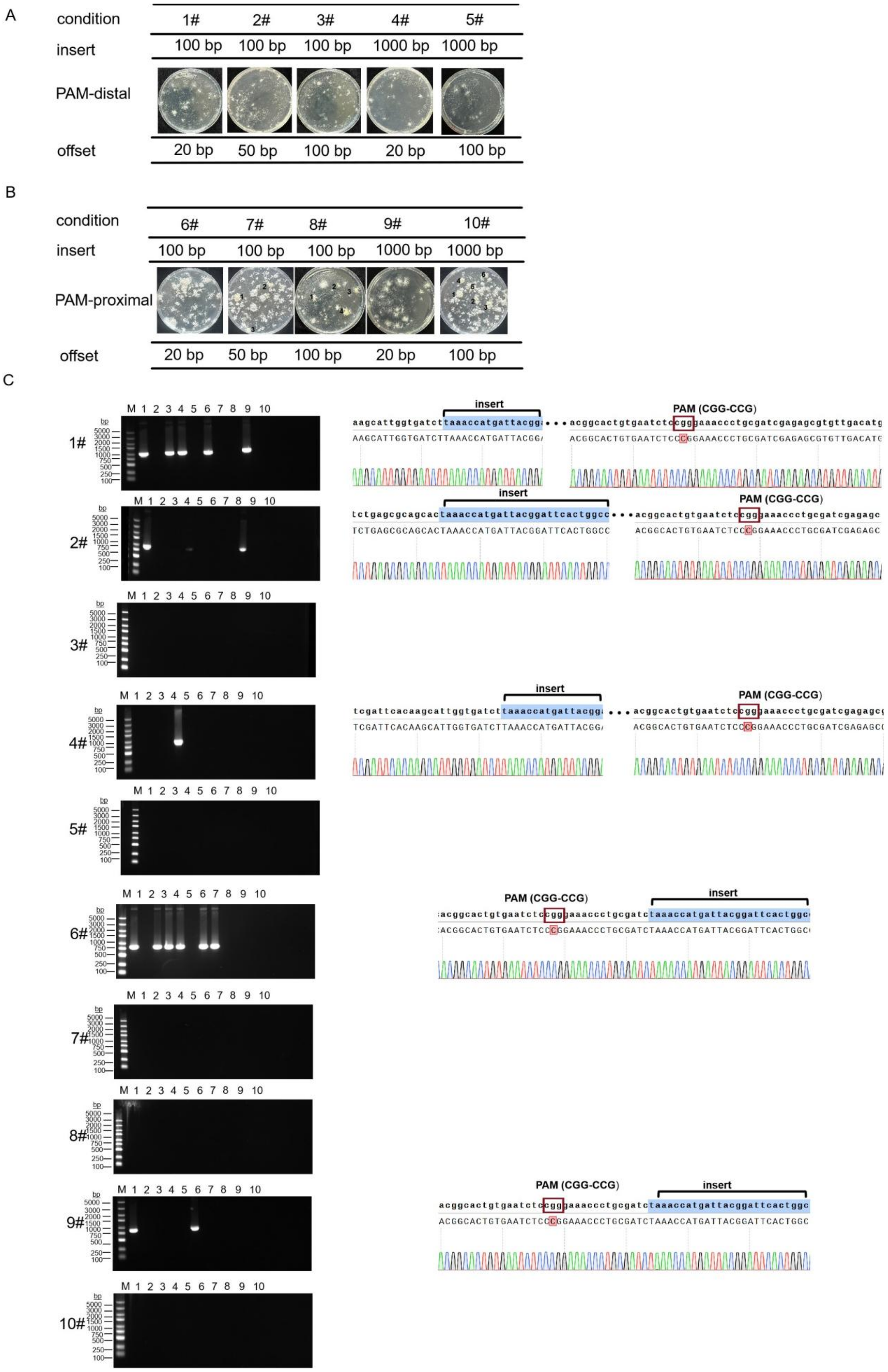

Supplementary Fig. S3. Validation of distance- and insert-length-dependent donor integration at the *wA* locus (Cas9 cut at +220 bp). Representative transformation plates and

colony PCR/Sanger sequencing validation for donor inserts positioned at defined distances from the Cas9 cleavage site. Each donor contained a 100-bp or 1-kb insert and was placed on either the PAM-distal or PAM-proximal side at offsets of 20, 50, or 100 bp. For each condition, ten colonies were screened using primers *check_F/check_R* (Supplementary Table 3). PCR-positive colonies were further confirmed by Sanger sequencing.

(A) Representative transformation plates for PAM-distal donor placements (Conditions #1–#5). The 100-bp donor was tested at −20, −50, and −100 bp, and the 1-kb donor at −20 and −100 bp relative to the Cas9 DSB.

(B) Representative transformation plates for PAM-proximal donor placements (Conditions #6–#10). The 100-bp donor was tested at +20, +50, and +100 bp, and the 1-kb donor at +20 and +100 bp relative to the DSB. Yellow colonies indicate potential insert-positive events.

(C) Colony PCR validation and representative Sanger sequencing chromatograms corresponding to Conditions #1–#10. The inserted donor sequence is shaded in blue; the PAM-disrupting synonymous mutation (CGG→CCG) is highlighted in red. Insert-positive PCR products were observed at −20, −50, and +20 bp, whereas no correct-integration colonies were detected at larger offsets, consistent with the distance-dependent efficiencies reported in Fig. 2D,E.

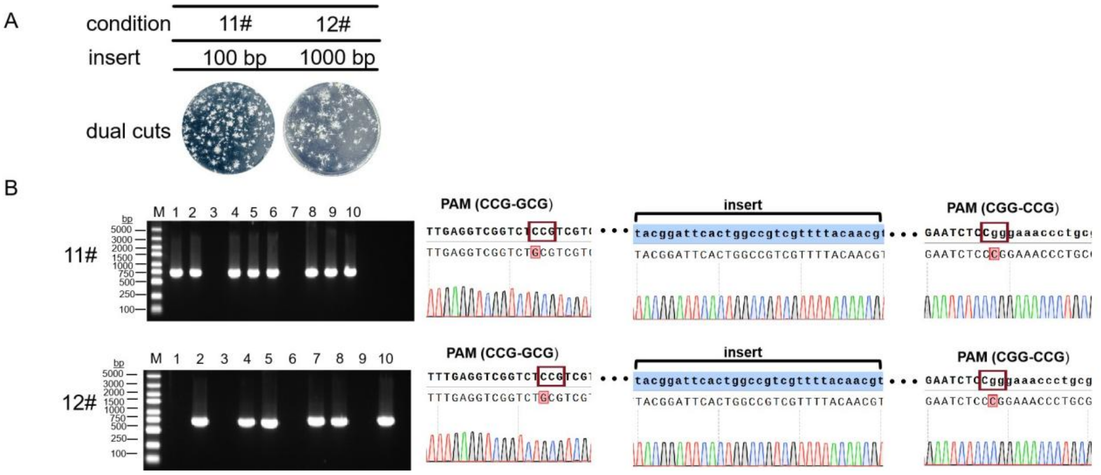

Supplementary Fig. S4. Length-dependent donor integration in the dual-DSB system at the *wA* locus (Cas9 cuts at +65 bp and +220 bp). Representative transformation plates, colony PCR, and Sanger sequencing validation for donor integration mediated by two Cas9 cleavage sites positioned at +65 bp (PAM-2) and +220 bp (PAM-1). Donors contained either a 100-bp or 1-kb insert flanked by 1-kb homology arms. For each condition, ten colonies were screened using primers *check_F/check_R* (Supplementary Table 3).

(A) Representative transformation plates for the dual-cut system using 100-bp (Condition #11) and 1-kb (Condition #12) donors.

(B) Colony PCR and representative Sanger sequencing for Conditions #11 and #12. Blue shading marks the inserted donor sequence; red boxes indicate PAM-disrupting synonymous mutations (CGG→CCG) introduced to prevent re-cleavage at both Cas9 sites. PCR-positive colonies confirm correct integration for both donor lengths, consistent with the efficiencies shown in Fig. 4B.

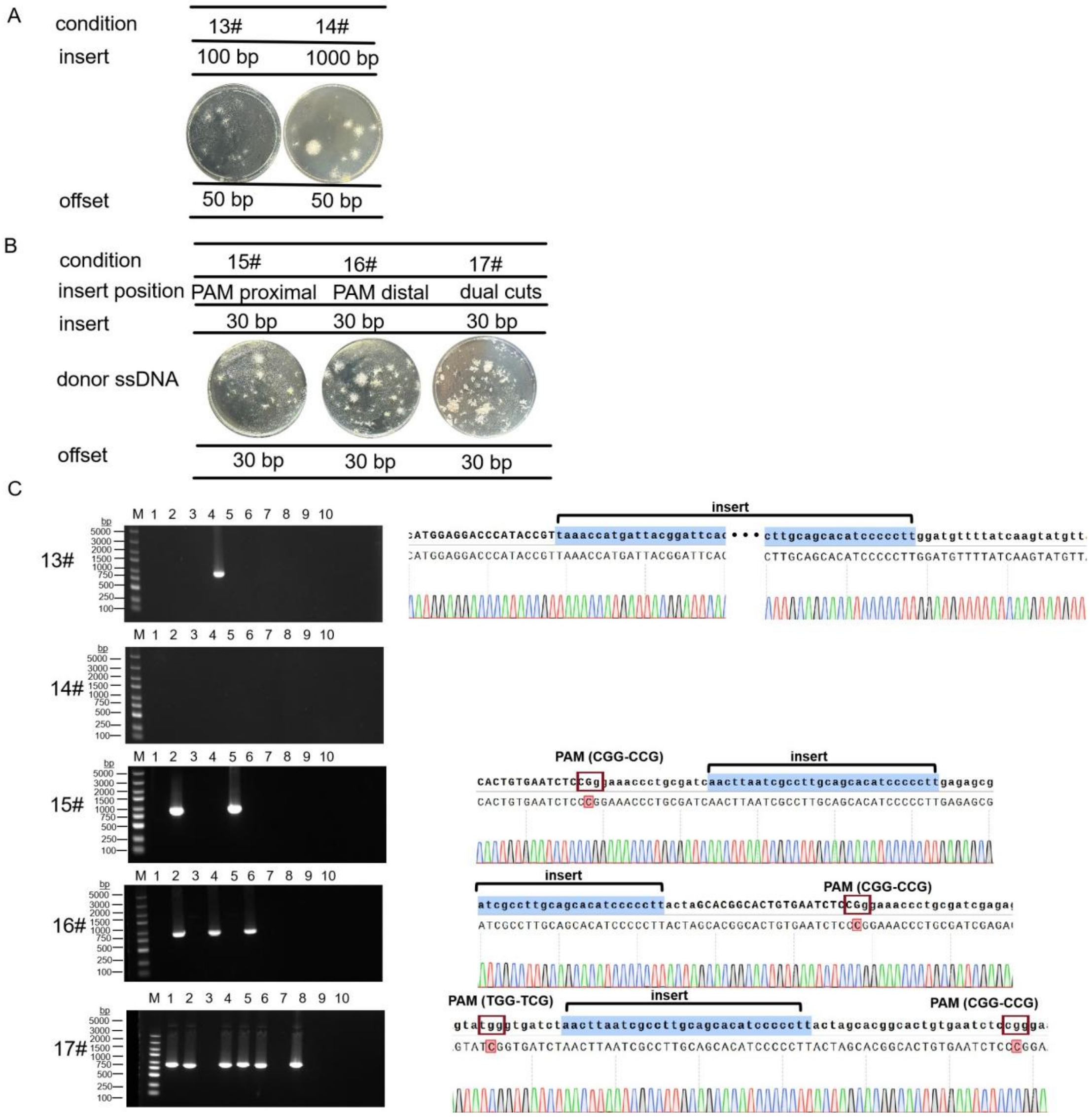

Supplementary Fig. S5. Donor-mediated integration at the *wA* locus of *A. nidulans*. Representative transformation plates and colony PCR/Sanger sequencing for dsDNA donors (conditions #13–14) and ssDNA donors (conditions #15–17). Ten colonies were examined for each condition using primers *check_F/check_R* (Supplementary Table 3); PCR-positive fractions are indicated. One of three independent experiments is shown.

(A) Transformation plates for dsDNA donor conditions #13 (100-bp donor; 50-bp offset from the +220-bp cut) and #14 (1-kb donor; 50-bp offset from the +220-bp cut).

(B) Transformation plates for ssDNA donor conditions #15–17 (30-bp insert with 30-bp homology arms; 30-bp offset). Condition #15: single-cut at +220 bp, PAM-proximal placement. Condition #16: single-cut at +220 bp, PAM-distal placement. Condition #17: dual-cut at +220/+65 bp.

(C) Colony PCR for conditions #13–17. For Sanger sequencing, one PCR-positive colony from each condition was selected as a representative clone. Blue shading marks the inserted

donor sequence; red boxes indicate PAM-disrupting synonymous mutations used to prevent re-cleavage. M, DNA ladder.Chromosomal DNA from putative edited strains was amplified by PCR, and the positive PCR products were directly subjected to DNA sequencing.

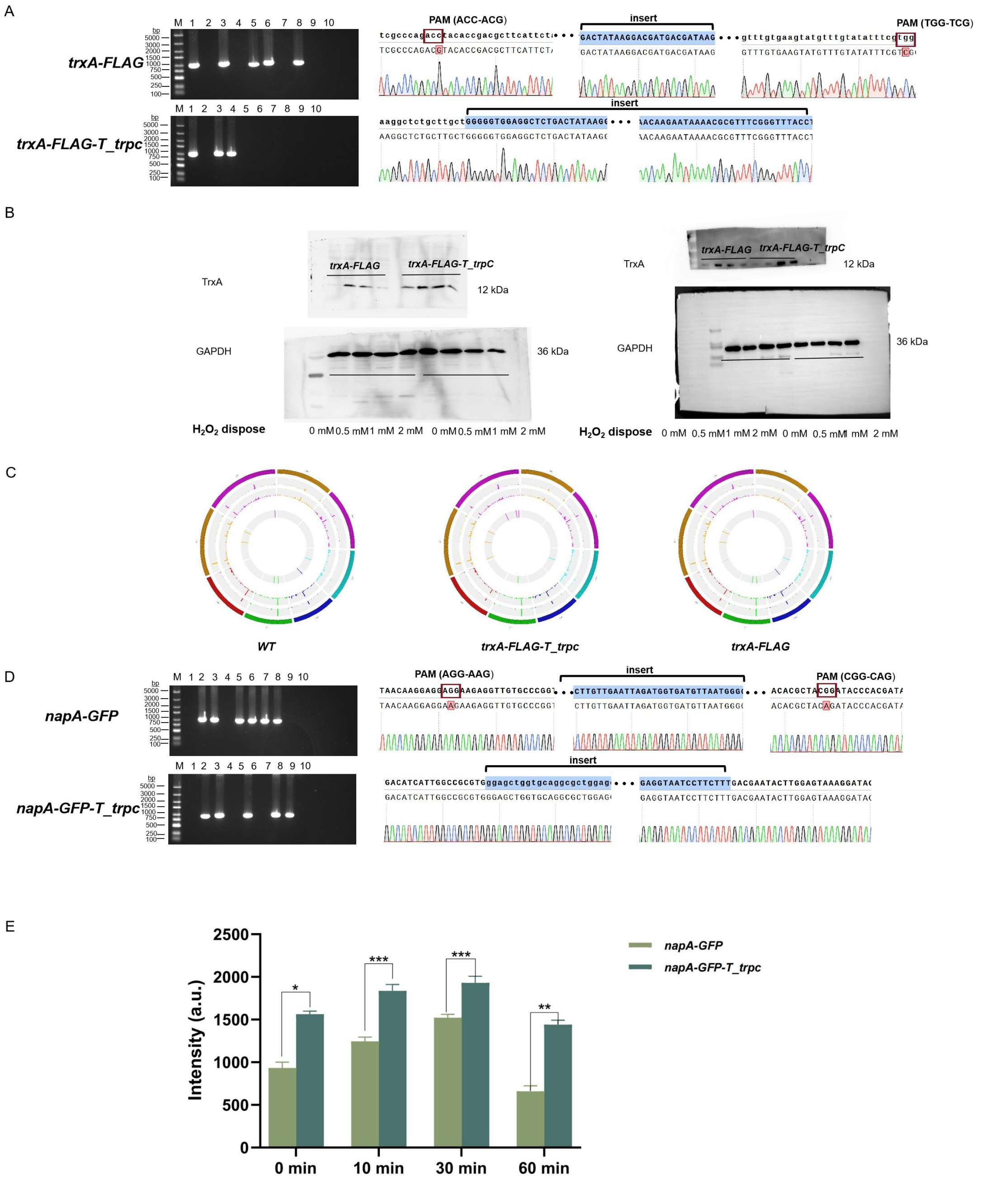

Supplementary Fig. S6. Validation of dual-sgRNA-mediated C-terminal tagging at the *trxA* and *napA* loci. All PCR gels and immunoblots shown were obtained from a single representative transformation. For colony PCR, ten transformants per condition were screened using primers listed in Supplementary Table 3. M, DNA ladder.

(A) Colony PCR confirming C-terminal *FLAG* insertion at the *trxA* locus using either a dual-sgRNA design (cut sites at −170 bp and +147 bp relative to the stop codon) or a single-sgRNA design (cut site at +147 bp). Representative Sanger sequencing traces of one PCR-positive clone per condition are shown.

(B) Western blot analysis of TrxA in strains carrying either *trxA-FLAG* or *trxA-FLAG-T_trpC* constructs, probed with anti-TrxA antibody. GAPDH was used as a loading control. Two additional biological replicates corresponding to Fig. 5E are shown.

(C) Circos plots illustrating genome-wide variant distribution in *WT*, *trxA-FLAG*, and *trxA-FLAG-T_trpC* strains. Tracks display chromosome layout, SNP density, InDel density, and structural variant distribution. All samples were processed using the same SV-calling, filtering, and plotting parameters. A small number of additional isolated SV signals are visible in the inner SV track of the *trxA-FLAG-T_trpC* strain. All variants were identified relative to the *A. nidulans* FGSC A4 reference genome; therefore, the *WT* control itself also contains background variation relative to the reference. In addition, the *trxA-FLAG* and *trxA-FLAG-T_trpC* strains were generated from *WT* stocks with different passage histories rather than from the same parental batch, which may contribute to background genetic differences among strains. Importantly, none of the detected SV breakpoints overlap with predicted CRISPR off-target loci (Supplementary Table S4, Sheet 3; SV "Off_target" annotations in Sheet 6, marked as "NO"), indicating that these variants are unlikely to result from CRISPR-induced off-target effects.

(D) Colony PCR confirming C-terminal *GFP* insertion at the *napA* locus using either a dual-sgRNA design (cut sites at −260 bp and +58 bp relative to the stop codon) or a single-sgRNA *GFP-T_trpC* donor. Representative Sanger sequencing traces of one PCR-positive clone per condition are shown.

(E) Quantification of GFP fluorescence in *napA–GFP* and *napA–GFP–T_trpc* strains. Hyphae were cultured in shaking flasks (MM liquid medium) for 12 h and then subjected to oxidative-stress treatment under the same conditions as used in Fig. 6C,D (1 mM H_2O_2 for 0, 10, 30, and 60 min). Mycelia were harvested by filtration, frozen in liquid nitrogen, and ground to a fine powder using a pre-chilled mortar and pestle. The powder was resuspended in extraction buffer and clarified by centrifugation. The supernatant was adjusted to 1 mg/mL total protein, and GFP fluorescence was measured using a fluorescence spectrophotometer (excitation/emission = 488/509 nm). Bars represent mean ± SD (n = 3), $^{*}P < 0.05$; $^{**}P < 0.01$; $^{***}P < 0.001$.

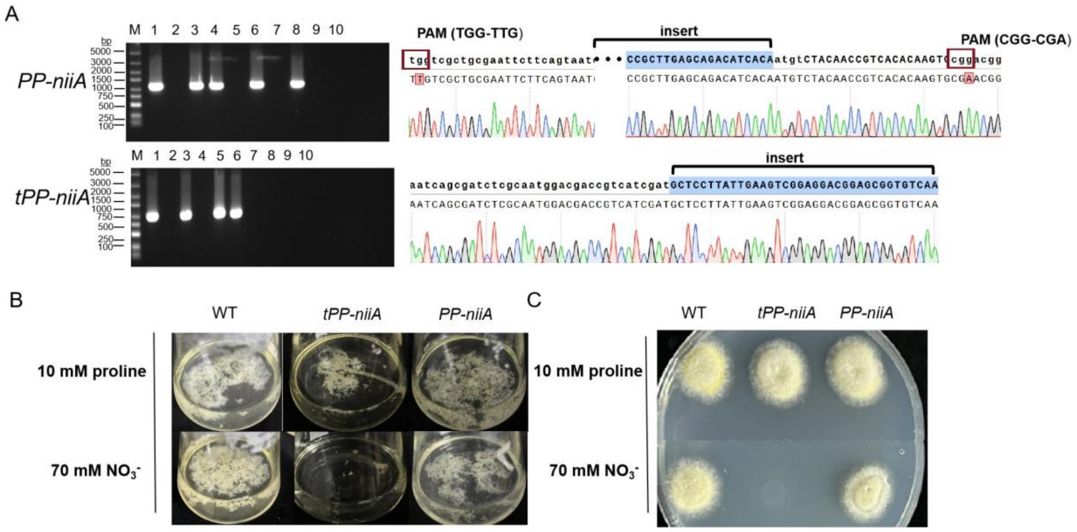

Supplementary Fig. S7. Validation of donor integration at the *niiA* locus using dual- and single-sgRNA strategies. Representative transformation plates, colony PCR, and Sanger sequencing for the edited *niiA* alleles. Ten colonies were screened for each editing condition using primers *niaD_check_F* / *niaD_check_R* for the *pp-niiA* strain and *niaD_control_check_F* / *niaD_control_check_R* for the *tpp-niiA* strain (Supplementary Table 3). For each condition, one PCR-positive colony was selected as a representative for sequencing. M, DNA ladder.

(A) Colony PCR verification of two edited configurations generated at the *niiA* locus. Conditions correspond to the constructs shown in Fig. 7A,B; PCR-positive fractions per condition are indicated below each gel.

(B) Growth of *WT*, *tPP-niiA*, and *PP-niiA* on solid medium containing proline or nitrate (NO_3^-) as the sole nitrogen source.

(C) Growth of the same strains in liquid culture under proline or nitrate (NO_3^-) conditions; representative images are shown.

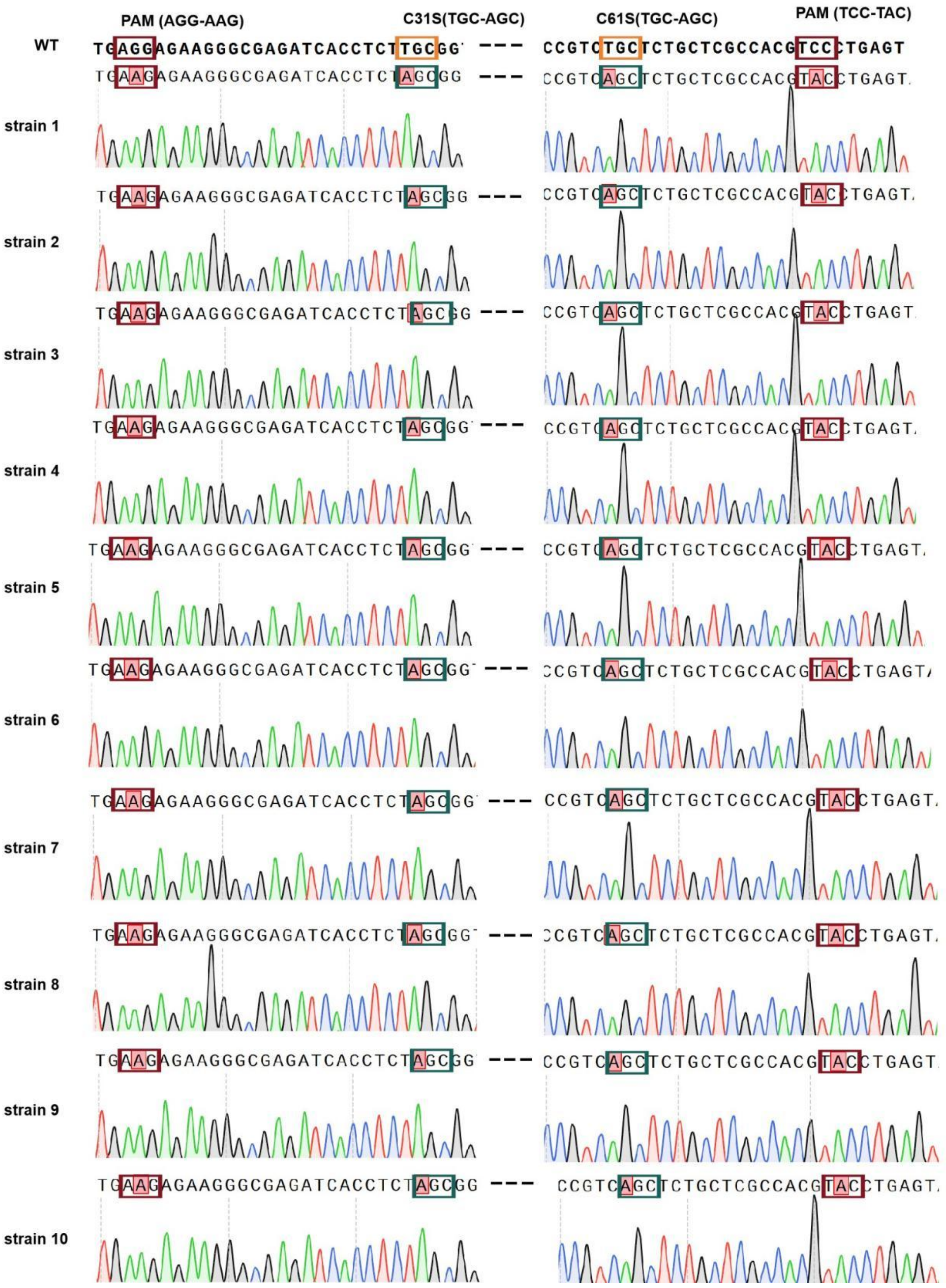

Supplementary Fig. S8. Validation of *prxA* double-point mutagenesis using a double-stranded DNA donor. Sanger sequencing chromatograms of ten independently isolated transformants generated

using the dual-sgRNA and dsDNA donor system. The donor template contained (i) a PAM-disrupting synonymous mutation (AGG→AAG) to block Cas9 re-cleavage and (ii) the intended nucleotide substitutions converting Cys31 and Cys61 to serine (C31S: TGC→AGC; C61S: TGC→AGC).
The left panel shows chromatograms covering the C31S region, and the right panel shows chromatograms covering the C61S region. Edited nucleotides are marked with green boxes, and the PAM-silencing mutation is indicated with red boxes.

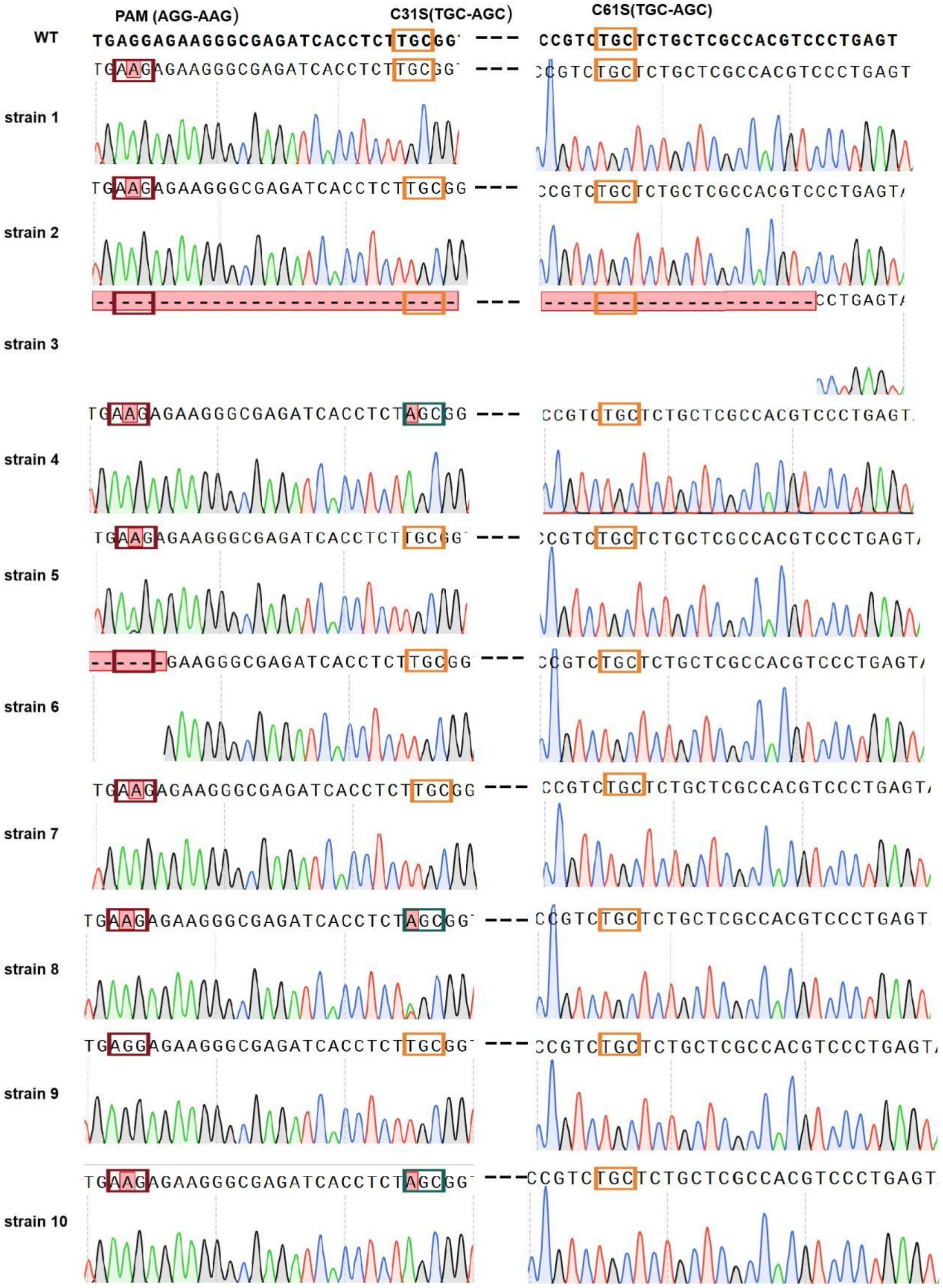

Supplementary Fig. S9. Validation of *prxA* point-mutation editing using an ssODN donor. Sanger sequencing chromatograms from 10 independent colonies generated using a single-cut ssODN donor. The ssODN donor encoded a PAM-disrupting synonymous substitution (AGG→AAG) and intended TGC→AGC changes at C31 and C61. Left panel:

region spanning the PAM and C31 site.Right panel: region spanning the C61 site. Red boxes mark the PAM-silent substitution; yellow shading indicates target nucleotides; green boxes denote intended edited bases when present.

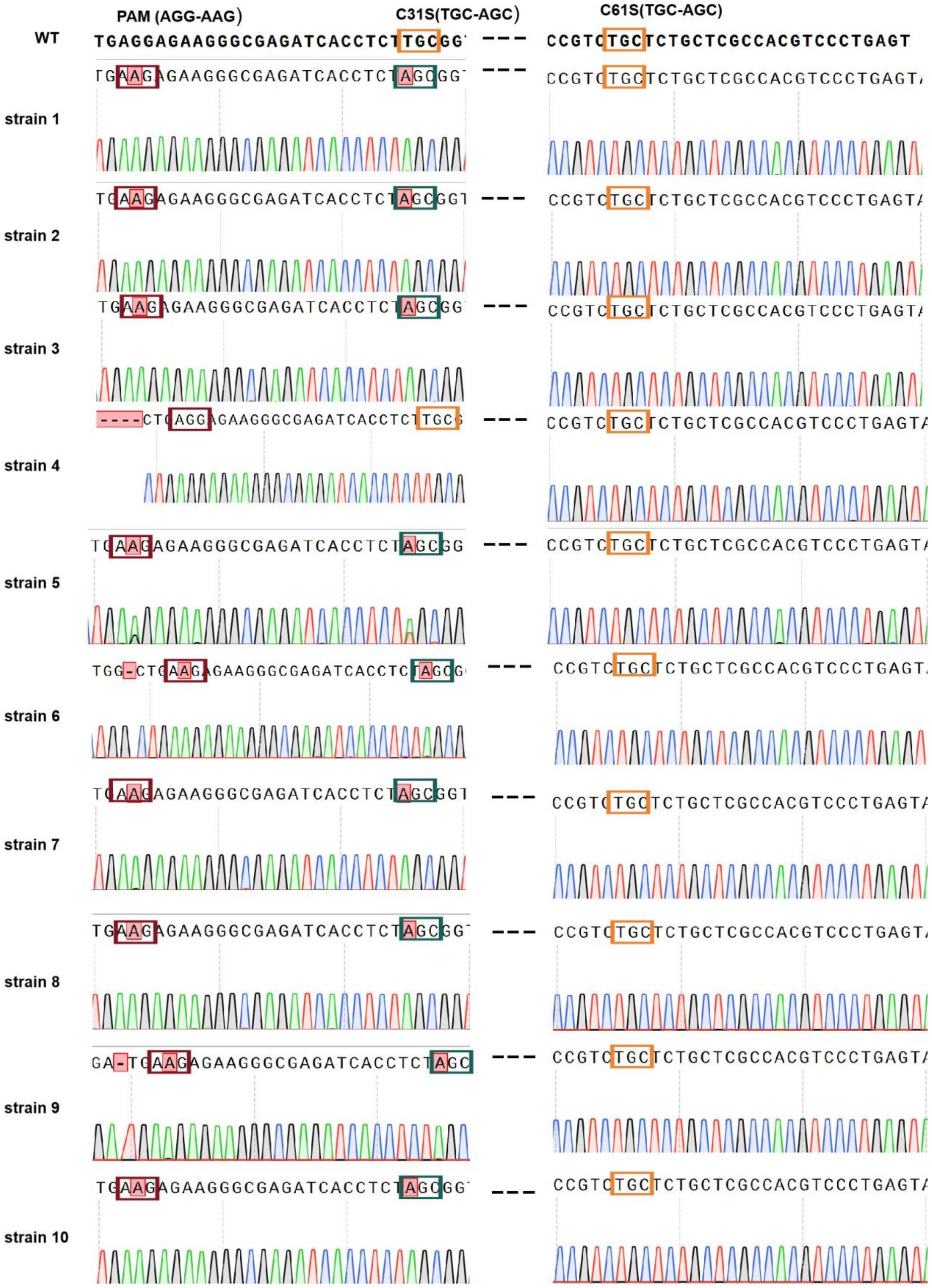

Supplementary Fig. S10. Validation of *prxA* double-site editing using a single-cut CRISPR design and a double-stranded DNA (dsDNA) donor. Sanger sequencing chromatograms from ten independently isolated colonies are shown. The single-cut donor design included a PAM-disrupting synonymous change (AGG→AAG) and two intended coding substitutions, C31S (TGC→AGC) and C61S (TGC→AGC). The chromatograms

display the regions surrounding the PAM and C31 sites (left) and the C61 site (right). Red boxes mark PAM-silencing mutations; yellow marks indicate the target nucleotide positions; green boxes highlight the bases corresponding to the intended edits.

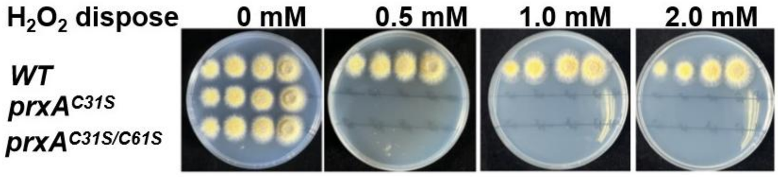

Supplementary Fig. S11. Growth assay of *prxA* point-mutation strains under hydrogen peroxide stress. Strains were spotted onto plates containing 0, 0.5, 1.0, or 2.0 mM H_2O_2 and incubated under standard conditions. Rows (top to bottom): *WT*, *prxA^C31S*, and *prxA^C31S/C61S*. One representative plate from three independent experiments is shown.

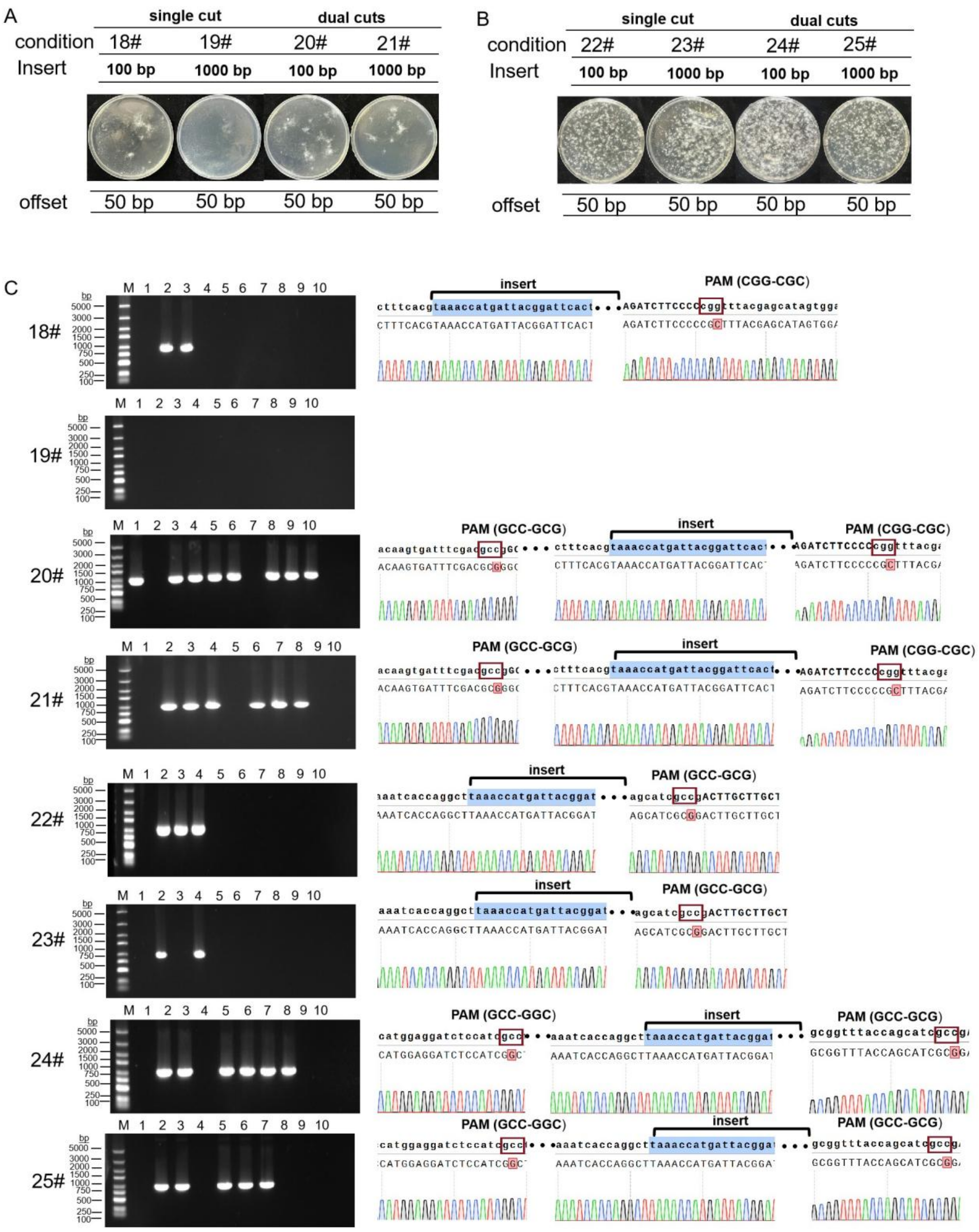

Supplementary Fig. S12. Donor integration at the *wA* locus of *A. oryzae* and the *abl1* locus of *A. fumigatus* under single- and dual-sgRNA configurations. Representative transformation plates and colony PCR/Sanger sequencing for conditions #18–25 using 100-bp or 1-kb dsDNA donors (1-kb homology arms; PAM-disrupting synonymous substitutions included). Ten colonies were examined for each condition using primers listed in Supplementary Table 3. M, DNA ladder.

(A) *A. oryzae*. Conditions #18–19: single-cut strategy with 100-bp and 1-kb donors (50-bp offset). Conditions #20–21: dual-cut strategy with 100-bp and 1-kb donors (50-bp offset).

(B) *A. fumigatus*. Conditions #22–23: single-cut strategy with 100-bp and 1-kb donors (50-bp offset). Conditions #24–25: dual-cut strategy with 100-bp and 1-kb donors (50-bp offset).

(C) Colony PCR and representative Sanger sequencing chromatograms for conditions #18–25. The inserted fragment is shaded in blue; PAM-disrupting synonymous changes are marked in red boxes.
